# Attenuation of reverse transcriptase facilitates SAMHD1 restriction of HIV-1 in cycling cells

**DOI:** 10.1186/s12977-023-00620-z

**Published:** 2023-05-01

**Authors:** Ming-Han C. Tsai, Sarah J. Caswell, Elizabeth R. Morris, Melanie C. Mann, Simon Pennell, Geoff Kelly, Harriet C. T. Groom, Ian A. Taylor, Kate N. Bishop

**Affiliations:** 1 Retroviral Replication Laboratory, The Francis Crick Institute, London, UK; 2 Macromolecular Structure Laboratory, The Francis Crick Institute, London, UK; 3 Structural Biology of DNA-Damage Signalling Laboratory, The Francis Crick Institute, London, UK; 4 The Medical Research Council Biomedical NMR Centre, The Francis Crick Institute, London, UK; 5 Present Address: LabGenius, London, UK; 6 Present Address: AstraZeneca, Granta Park, Cambridge, UK; 7 Present Address: Department of Biosciences, University of Durham, Durham, UK; 8 Present Address: Sartorius, Ulm, Germany; 9 Present Address: MRC London Institute of Medical Sciences, London, UK; 10 Present Address: Department of Medicine, University of Cambridge, Cambridge, UK

**Keywords:** SAMHD1, HIV-1, Restriction, dNTP levels, Phosphorylation, Cycling cells

## Abstract

**Background:**

SAMHD1 is a deoxynucleotide triphosphohydrolase that restricts replication of HIV-1 in differentiated leucocytes. HIV-1 is not restricted in cycling cells and it has been proposed that this is due to phosphorylation of SAMHD1 at T592 in these cells inactivating the enzymatic activity. To distinguish between theories for how SAMHD1 restricts HIV-1 in differentiated but not cycling cells, we analysed the effects of substitutions at T592 on restriction and dNTP levels in both cycling and differentiated cells as well as tetramer stability and enzymatic activity in vitro.

**Results:**

We first showed that HIV-1 restriction was not due to SAMHD1 nuclease activity. We then characterised a panel of SAMHD1 T592 mutants and divided them into three classes. We found that a subset of mutants lost their ability to restrict HIV-1 in differentiated cells which generally corresponded with a decrease in triphosphohydrolase activity and/or tetramer stability in vitro. Interestingly, no T592 mutants were able to restrict WT HIV-1 in cycling cells, despite not being regulated by phosphorylation and retaining their ability to hydrolyse dNTPs. Lowering dNTP levels by addition of hydroxyurea did not give rise to restriction. Compellingly however, HIV-1 RT mutants with reduced affinity for dNTPs were significantly restricted by wild-type and T592 mutant SAMHD1 in both cycling U937 cells and Jurkat T-cells. Restriction correlated with reverse transcription levels.

**Conclusions:**

Altogether, we found that the amino acid at residue 592 has a strong effect on tetramer formation and, although this is not a simple “on/off” switch, this does correlate with the ability of SAMHD1 to restrict HIV-1 replication in differentiated cells. However, preventing phosphorylation of SAMHD1 and/or lowering dNTP levels by adding hydroxyurea was not enough to restore restriction in cycling cells. Nonetheless, lowering the affinity of HIV-1 RT for dNTPs, showed that restriction is mediated by dNTP levels and we were able to observe for the first time that SAMHD1 is active and capable of inhibiting HIV-1 replication in cycling cells, if the affinity of RT for dNTPs is reduced. This suggests that the very high affinity of HIV-1 RT for dNTPs prevents HIV-1 restriction by SAMHD1 in cycling cells.

**Supplementary Information:**

The online version contains supplementary material available at 10.1186/s12977-023-00620-z.

## Background

Sterile α-motif and histidine-aspartate domain-containing protein 1 (SAMHD1) is a deoxynucleotide triphosphohydrolase [1]. Human SAMHD1 is a 626-amino-acid protein, which contains an N-terminal nuclear localisation signal [2], a SAM domain that in other systems mediate protein–protein interactions [3, 4] and an HD catalytic domain followed by a C-terminal region [1, 5–8]. Together with ribonuclease reductase (RNR), SAMHD1 is responsible for dNTP homeostasis in cells [6, 9]. In 2011, SAMHD1 was identified as a restriction factor of HIV-1 in cells of the myeloid-lineage and resting CD4 + T cells [10–14]. The HIV-2 encoded accessory protein, Vpx, is able to overcome SAMHD1-mediated restriction by targeting SAMHD1 for degradation via the proteasome [15, 16]. It was proposed that SAMHD1 restricts HIV-1 in these cells by reducing cellular dNTP levels to below the threshold that is required for viral reverse transcription [1, 17, 18]. Accordingly, SAMHD1 restriction was not observed in cycling or activated cells which typically have higher dNTP levels [7, 19].

It has become evident that the formation of a stable GTP-dNTP activated SAMHD1 tetramer is optimal for catalysing the hydrolysis of dNTPs [5, 20–23], and that the phosphorylation of SAMHD1 at Threonine 592 (T592) destabilises the tetramer [5, 24–26]. During the cell cycle, SAMHD1 is regulated through phosphorylation by different cyclin-dependent kinases (CDKs) and cyclins. DNA replication in cycling cells needs dNTP substrates, requiring reduced dNTP turnover, and from late G1 to S phase the majority of SAMHD1 is phosphorylated through the activities of CDK6 and CDK2 coupled with Cyclins E and A respectively [27, 28]. In G2 and M phases, SAMHD1 is also largely phosphorylated but by CDK1 coupled with Cyclin A2 [19]. By contrast, at the end of M phase after cell division has concluded, SAMHD1 is dephosphorylated by the PP2A-B55α phosphatase [29] prior to entry into G1 or a differentiated/quiescent state (G0). In addition, SAMHD1 expression levels vary both with cell type and also in the different stages of cell cycle [30]. In particular, increased SAMHD1 expression is found in quiescent primary cells and terminally differentiated immune cells which maintain low levels of cellular dNTPs [6, 28, 31].

The current model in the field suggests that phosphorylation of SAMHD1 attenuates its enzyme activity, resulting in decreased dNTP hydrolysis and therefore higher dNTP levels in cycling cells, which can then support reverse transcription [17–19, 28, 32, 33]. In agreement with this hypothesis, phosphomimetic mutants of SAMHD1 were unable to restrict HIV-1 in differentiated cells [7, 33, 34]. However, there is growing evidence from in vitro experiments that phosphorylated SAMHD1 can still maintain catalytic function if dNTP levels are high [5, 7, 34, 35], suggesting that the regulation of SAMHD1 activity is not solely through phosphorylation. In addition, alternative hypotheses for how SAMHD1 restricts HIV-1 have been proposed, including RNA binding and RNA degradation [36–44].

To distinguish between the various theories for HIV-1 restriction by SAMHD1 and to understand why SAMHD1 fails to restrict HIV-1 in cycling cells, we have analysed the effects of substitutions at T592 on both restriction and dNTP levels in both cycling and differentiated cells as well as on tetramer stability and enzymatic activity in vitro. First, we showed that viral genomes remain intact in the presence of SAMHD1 and are competent to replicate once SAMHD1 is removed, arguing against the notion that SAMHD1 nuclease activity is responsible for restriction. This was supported by the lack of detectable nuclease activity in vitro. We then tested a panel of T592 mutants and showed that a subset had lost their ability to restrict HIV-1 in differentiated cells which generally correlated with a decrease in triphosphohydrolase activity and/or tetramer stability in vitro. However, we were unable to induce restriction in cycling cells by preventing phosphorylation of SAMHD1 until we reduced the affinity of HIV-1 RT for dNTPs. We were then able to observe for the first time that SAMHD1 is an active deoxynucleotide triphosphohydrolase and is capable of inhibiting HIV-1 replication in cycling cells. This suggests that the very high affinity of HIV-1 RT for dNTPs prevents HIV-1 restriction by SAMHD1 in cycling cells and implies that dNTP levels in differentiated cells are far below the level of detection by typical dNTP assays. Altogether, our work supports the idea that HIV-1 RT has evolved to function at lower dNTP levels due to a lack of SAMHD1-targeting accessory proteins in HIV-1, emphasising that the main function of Vpx is to counteract the dNTPase activity of SAMHD1, rather than any nuclease activity.

## Results

### Vpx can rescue HIV-1 replication up to 48 h post infection

**Fig. 1:**
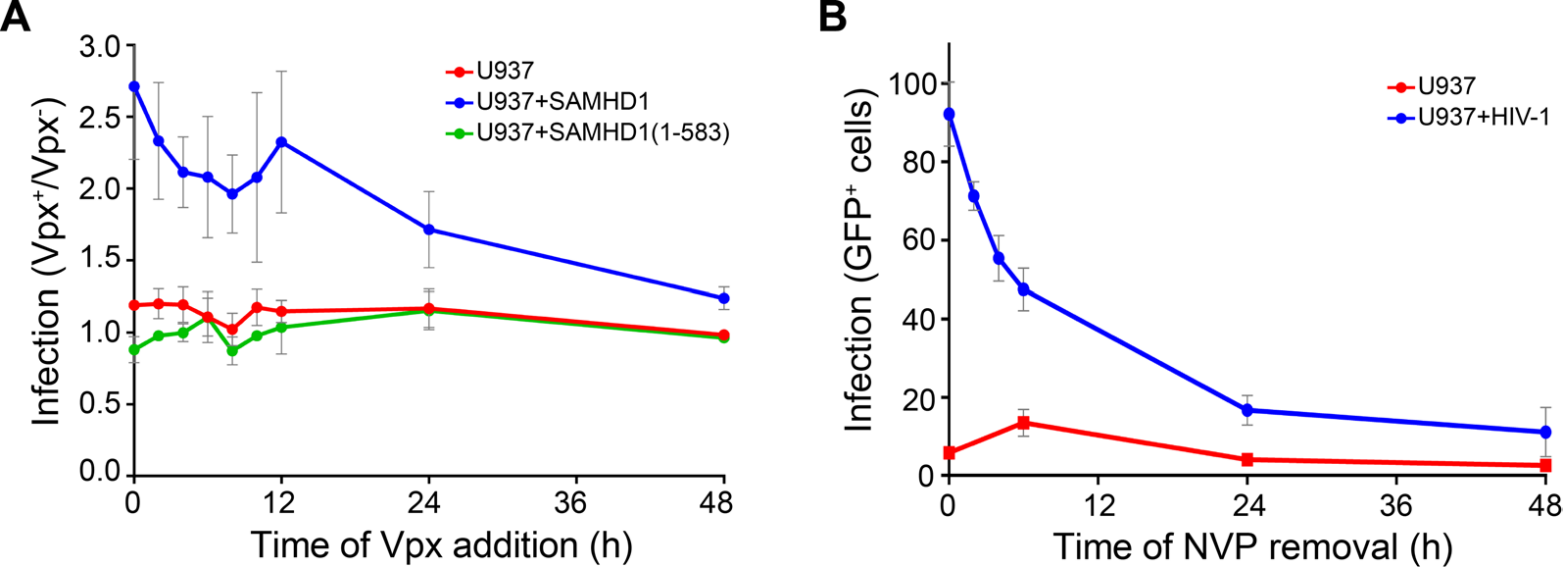
Time dependence of Vpx rescue of HIV-1 infection (**A**) Time course of Vpx rescue of HIV-GFP infection. Vpx was introduced into either differentiated parental U937 cells (red line) or cells expressing WT SAMHD1 (Blue line) or the inactive SAMHD1(1–583) mutant (green line), co-incident with HIV-GFP or at intervals up to 48 h post-infection. Cells were harvested at 96 h post-infection and analysed for GFP expression by flow cytometry. The plot shows the ratio of Vpx^+^ cells expressing GFP relative to Vpx^−^ cells expressing GFP, error bars represent the range of at least three independent experiments. A ratio of one indicates that the presence of Vpx had no effect on HIV-GFP infection. **B** Nevirapine washout assay. Differentiated U937 cells were treated with media (red line) or media containing 10 µM Nevirapine (blue line) before infection with HIV-GFP. At the indicated time post-infection, the media was changed to remove the drug. Cells were harvested at 96 h post-infection and analysed for GFP expression using flow cytometry. The percentage of GFP^+^ cells are plotted, error bars represent the range of at least three independent experiments

Although SAMHD1 has a clearly demonstrated deoxynucleotide triphosphohydrolase activity that is required for HIV-1 restriction [1], it has also been proposed that it can restrict HIV by binding to and/or degrading the viral RNA genome [39]. If restriction did occur by genome degradation, it would be expected to be irreversible. Therefore, Vpx would only prevent restriction, by promoting SAMHD1 degradation, if it was present before SAMHD1 encountered the viral RNA. To investigate this, we employed a “Vpx rescue” assay to measure how long after infection the introduction of Vpx could relieve restriction by SAMHD1 and rescue HIV-1 replication. We infected U937 cells, which do not express SAMHD1, or U937 cells stably expressing WT SAMHD1 or SAMHD1(1–583), a mutant that we have previously shown is deficient in HIV-1 restriction [5], with HIV-1 VLPs encoding GFP. We then provided Vpx *in trans* at different times post infection by infecting with SIVmac VLPs containing WT Vpx (Vpx^+^) or without Vpx (Vpx^−^) as the control (Additional file 1: Figure S1). GFP expression was measured after 48 h, and an infection ratio was enumerated as the proportion of GFP positive cells in the Vpx^+^ population compared to the proportion of GFP positive cells in the Vpx^−^ population. The results of this Vpx rescue assay are presented in Fig. 1A. The presence of Vpx had little effect on GFP expression in infected U937 cells (red line) or U937-SAMHD1(1–583) cells (green line) resulting in an infection ratio of approximately 1.0 over the whole time-course. However, in U937 cells expressing WT SAMHD1 (blue line), the addition of Vpx^+^ SIV-VLPs considerably increased HIV-1 replication and this effect was still apparent when Vpx was added 48 h after infection. Addition of Vpx within the first 12 h post infection resulted in two–threefold higher GFP expression and was still 1.5-fold when Vpx was added 48 h post infection. Therefore, these data suggest that an intact HIV genome must still be present even after 48 h exposure to SAMHD1 and support the notion that SAMHD1-suppression of the dNTP pool rather SAMHD1-mediated degradation of the HIV-1 genome is the cause of the block to infection. However, the ability of Vpx to rescue HIV-1 infection does decrease with time, most likely due to degradation/clearance of stalled replication complexes. Therefore, to test this and rule out any late acting SAMHD1 nuclease activity, we used Nevirapine to block HIV-1 replication in U937 cells and monitored the recovery of infection following its removal at various times (Fig. 1B). In this assay, the capacity of HIV-1 VLPs to express GFP was reduced by around 90% after 48 h of Nevirapine treatment and closely mirrored the reduction in the ability of Vpx to rescue infection. Therefore, the observed decrease in the ability of Vpx to rescue infection with time is likely independent of SAMHD1. This observation is supported by our in vitro experiments that showed no nuclease activity against a range of substrates (Additional file 1: Figure S2). Figure S2E shows that although partially purified preparations of SAMHD1 (affinity-purified only and affinity-purified followed by size exclusion chromatography) show some spurious nuclease activity against single stranded RNA (ssRNA), if highly purified preparations of SAMHD1 were employed, nuclease activity becomes undetectable (Additional file 1: Figure S2E).

### SAMHD1 T592 mutants can be categorised as stable or unstable tetramers

After eliminating a SAMHD1 nuclease activity as the restriction mechanism, we next wanted to investigate whether reduced triphosphohydrolase activity alone could explain the lack of HIV-1 restriction by SAMHD1 in cycling cells. Phosphorylation of T592 has been proposed to downregulate SAMHD1 hydrolase activity in cycling cells through destabilisation of the SAMHD1 tetramer [5, 24, 34]. We therefore generated a panel of substitution mutants at the T592 phosphorylation site that included the previously reported phosphomimetics, T592D and T592E, and the aliphatic T592A and T592V substitution mutants [5, 7, 19, 28, 33]. In addition, we also made the phospho-null mutants T592C, T592K, T592I and T592L and the potential phospho-substrate mutant T592S. These mutants (Additional file 1: Figure S3A) were chosen in an effort to modulate the stability of SAMHD1 tetramerisation by choosing residues that might destabilise tetramerisation through phosphomimetic (T592D and T592E) or steric effects (T592K, T592L), remain neutral (T592A, T592S) or potentially stabilise tetramerisation through favourable side-chain packing of the β-methyl or sulfhydryl groups but be refractory to further phosphorylation (T592V, T592I and T592C) (Additional file 1: Figure S3B). All the mutants were expressed at a similar level to WT SAMHD1 when transduced into U937 cells (Additional file 1: Figure S3C, D), but only WT SAMHD1 and the active site dead mutant HD206/207(AA) showed any phosphorylation as detected by immunoblotting (Additional file 1: Figure S3C).

**Fig. 2:**
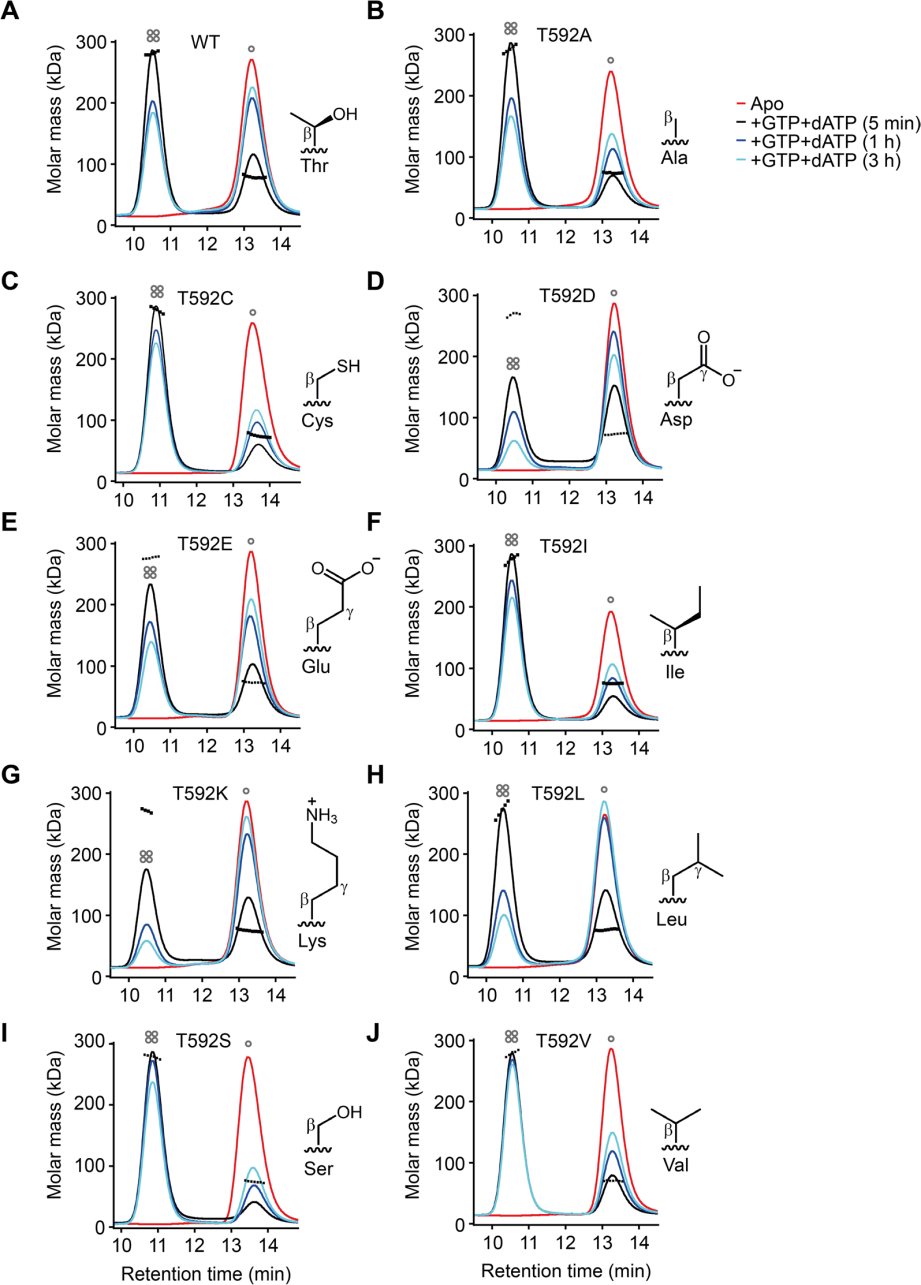
Tetramer stability of T592 mutants SEC–MALLS analysis of tetramer stability for (**A**) WT SAMHD1 and (**B**–**J**) SAMHD1 T592 mutants after addition of GTP and dATP nucleotides. The solid lines are the chromatograms from the output of the differential refractometer and the black scatter points are the weight-averaged molar masses determined at 1 s intervals throughout elution of chromatographic peaks, SAMHD1 monomer-dimers elute at 13.0–14.0 min, tetramers at 10.5–11.0 min. The displayed chromatograms in each panel are (red) apo-SAMHD; (black) SAMHD1 and 0.2 mM GTP, 0.5 mM dATP after 5 min incubation; (blue) SAMHD1 and 0.2 mM GTP, 0.5 mM dATP after 60 min incubation; (cyan) SAMHD1 and 0.2 mM GTP, 0.5 mM dATP after 180 min incubation. The side chain configuration of the amino acid substitutions at T592 are displayed adjacent to the chromatograms

We first assessed the in vitro stability of T592 mutant tetramers using Size Exclusion Chromatography-Multi-Angle Laser Light Scattering (SEC–MALLS). Although conditions inside cells may affect overall SAMHD1 stability, this method allowed us to look at the intrinsic stability of the mutant proteins. Tetramer assembly reactions were initiated by addition of GTP and dATP and the fraction of tetramer was measured at increasing intervals of up to 3 h after nucleotide addition. Figure 2 shows SEC–MALLS data from which tetramer decay rates (*k*_*dec*_) were calculated. Table 1 shows that T592A, T592C, T592I, T592S and T592V have slower decay rates than WT SAMHD1, and T592D, T592K and T592L have faster *k*_*dec*_. These data broadly agree with calculations from the SAMHD1 structure (Additional file 1: Figure S3B) that suggest that whilst short Cβ branched residues would stabilise packing around residue 592 in the C-terminal lobe, larger side chains and charged residues would be destabilising. Thus phosphorylation, being both bulky and charged, would be expected to inhibit tetramerisation in a similar fashion. We then measured catalytic turnover (*k*_*cat*_) for GTP stimulated hydrolysis of dATP for each mutant using ^1^H NMR (Additional file 1: Figure S4 and Table 1). These data show that all T592 mutants can hydrolyse dATP under these steady state conditions, with *k*_*cat*_ seven–tenfold lower than that of WT SAMHD1, with the exception of T592S that had a 37-fold lower *k*_*cat*_. An inherently faster *k*_*cat*_ may affect tetramer stability in SEC–MALLS experiments due to depletion of allosteric activators. Therefore, to compare the stability of WT SAMHD1 and each T592 mutant, we amalgamated the capacity to stabilise tetramers and hydrolyse dNTPs into the combined parameter of *(k*_*cat*_*/k*_*dec*_*)*. The application of this index revealed that whilst none of the T592 mutants appear to be as overall effective as WT SAMHD1, the mutants fall into three classes, Class-1 (Tet^stable^) those with *k*_*cat*_*/k*_*dec*_ only two–fourfold lower than WT (T592C, T592I and T592V), Class-2 (Tet^inter^) those with *k*_*cat*_*/k*_*dec*_ six–eightfold lower than WT (T592A and T592E) and Class-3 (Tet^unstable^) those with *k*_*cat*_*/k*_*dec*_ > 12 fold lower than WT (T592D, T592K, T592L and T592S).

**Table 1:** SAMHD1 catalytic turnover and tetramer stability of T592 mutants

SAMHD1	Substrate	AL1	AL2	*k*_*cat*_ (s^−1^)	*f*_*tet*_^*b*^* (3 h)*	*(k*_*dec*_*)*^*c*^*(s*^*−1*^*)* × *10*^*5*^	*(k*_*cat*_*/k*_*dec*_*)*
*w.t*	dATP	GTP	dATP	0.864 ± 0.092^a^	0.38	7.02	12,308
T592A	dATP	GTP	dATP	0.087 ± 0.029	0.50	5.14	*1692*
T592C	dATP	GTP	dATP	0.096 ± 0.021	0.63	2.94	3265
T592D	dATP	GTP	dATP	0.100 ± 0.012	0.19	11.2	***893***
T592E	dATP	GTP	dATP	0.111 ± 0.018	0.36	7.00	*1586*
T592I	dATP	GTP	dATP	0.101 ± 0.009	0.63	3.07	3289
T592K	dATP	GTP	dATP	0.120 ± 0.019	0.12	22.1	***543***
T592L	dATP	GTP	dATP	0.127 ± 0.042	0.20	15.2	***836***
T592S	dATP	GTP	dATP	0.023 ± 0.004	0.68	2.33	***987***
T592V	dATP	GTP	dATP	0.104 ± 0.025	0.61	2.53	4111

^a^Error is the SEM of at least three independent measurements

^b^Fraction tetramer remaining after 3 h incubation

^c^Tetramer decay rate determined from fitting the f_tet_ between 5 min and 3 h to an exponential decay function Y = Ae^−kdec.x^. *k*_*cat*_*/k*_*dec*_ values are grouped as WT to WT/4 (plain text), WT/6 to WT/8 (Italics) and < WT/12 (Bold-italics)

### Stable T592 mutants do not restrict HIV-1 in cycling U937 cells despite reducing dNTP levels

**Fig. 3:**
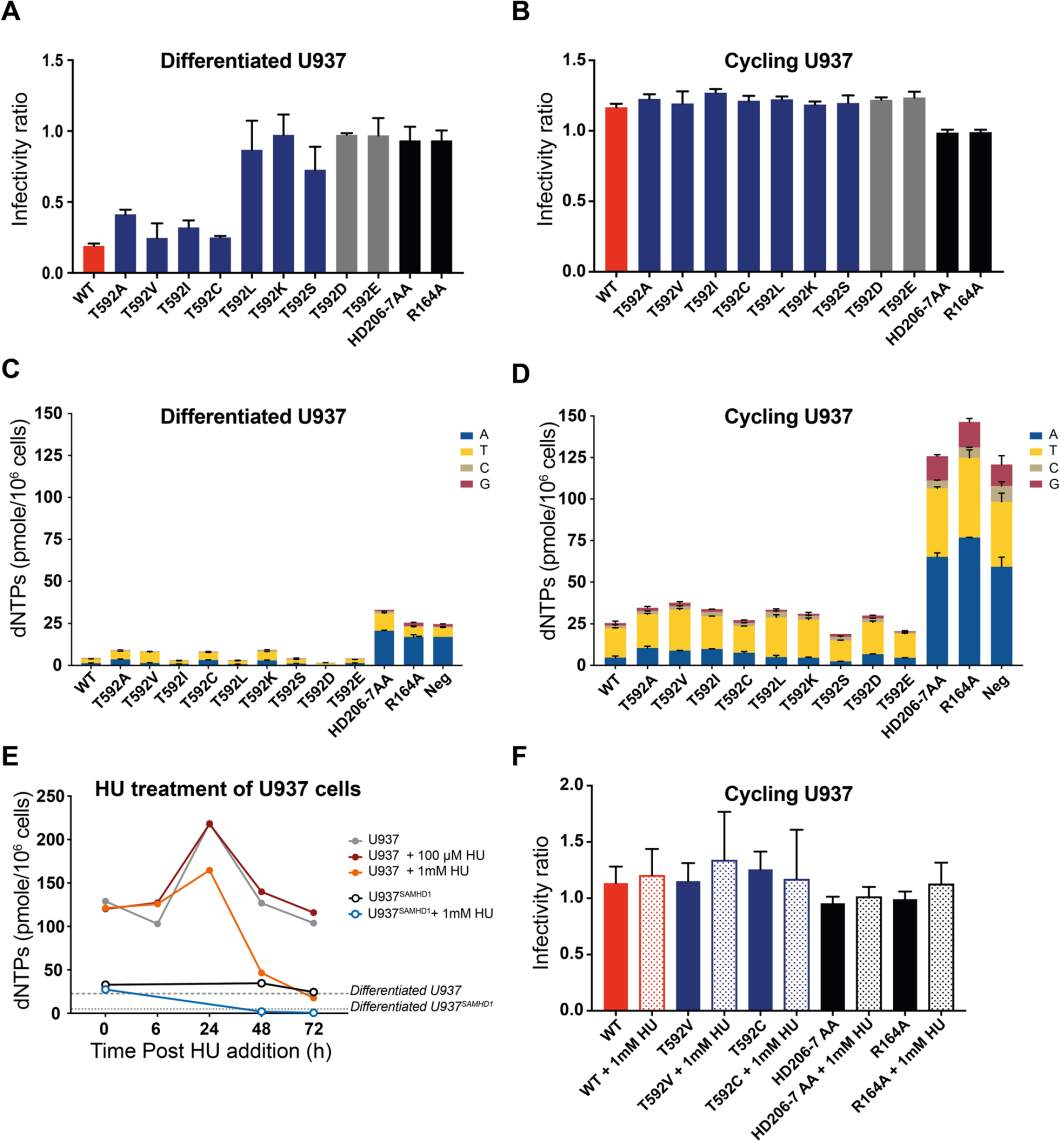
HIV-1 restriction and dNTP levels in U937 cells expressing SAMHD1 T592 mutants (**A** and **B**) HIV-1 restriction assay in (**A**) differentiated and (**B**) cycling U937 cells. Cells were first transduced with a bicistronic construct expressing WT SAMHD1 or the indicated mutant and YFP, followed by infection with HIV-GFP. Cells were harvested after 72 h and analysed by flow cytometry. The infectivity ratio was calculated by dividing the percentage of GFP positive cells in the YFP (SAMHD1) positive population by the percentage of GFP positive cells in the YFP (SAMHD1) negative population. The bars show the average of three biological repeats and error bars represent the SD. The T592 mutants, phosphomimetic mutants, and inactive negative controls are shown in blue, grey, and black, respectively. **C** and **D** Measurements of dNTP levels from (**C**) differentiated and (**D**) cycling U937 cells expressing SAMHD1 and indicated mutants (Neg = untransduced U937 cells). The amount of dATP, TTP, dCTP, and dGTP within the total bar stack are shown in blue, yellow, beige, and red. The values are an average of two technical repeats and error bars represent the SEM. **E** The effect of HU treatment on the levels of dNTPs. Cycling parental U937 (solid circles), or WT SAMHD1 transduced, U937^SAMHD1^ (open circles) were treated with 100 µM (red line) or 1 mM (orange and blue lines) HU or untreated (grey and black lines) and samples were taken for dNTP analysis at time points up to 72 h after addition of HU. Dashed and dotted grey thresholds indicate the dNTP levels measured for untreated differentiated U937 and U937^SAMHD1^ cells respectively. Each point represents the average sum of all four nucleotides (pmole per 10^6^ cells) from two technical repeats. **F** HIV-1 Restriction assay in cycling U937 cells transduced with WT SAMHD1 (red bars), T592V or T592C mutants (blue bars) or catalytically inactive HD206-7AA and R164A controls (black bars) following 1 mM HU treatment for 72 h (dotted bars) or untreated (solid bars). Restriction assay was performed as above. The bars are the average of three biological repeats and error bars represent the SD

Having identified a class of SAMHD1 T592 non-phosphorylatable Tet^stable^ mutants with close to WT tetramer properties (T592C/I/V) and a set of Tet^unstable^ mutants (T592/D/K/L) as well as the catalytically deficient T592S, we then asked what effect tetramer stabilisation had on HIV-1 restriction in cycling and differentiated U937 cells. Each SAMHD1 mutant was transduced into U937 cells as a bicistronic construct that also expressed YFP and half the cells were then differentiated by PMA treatment. Following infection with an HIV-1-GFP vector, HIV-1 restriction was measured using two-colour flow cytometry and an infectivity ratio was calculated by dividing the percentage of SAMHD1 positive cells infected with HIV-1 by the percentage of SAMHD1 negative cells infected with HIV-1. Thus, an infectivity ratio < 1 indicates restriction by SAMHD1. These data, Fig. 3A, show that the Tet^stable^ group (T592/C/I/V) along with T592A from the Tet^inter^ group had similar levels of restriction to WT SAMHD1 in differentiated U937 cells (infectivity ratio of ~ 0.2), while the Tet^unstable^ group (T592/D/K/L), T592S and the other phosphomimetic mutation T592E showed no restriction, as did catalytically dead negative control mutants, HD206-7AA and R164A. These data support previous observations that destabilisation of SAMHD1 tetramers as well as impairment of triphosphohydrolase activity prevents HIV-1 restriction in differentiated cells [5, 21, 24, 34]. As observed previously, WT SAMHD1 showed no restriction of HIV-1 infection in cycling U937 cells (Fig. 3B) and neither did the Tet^unstable^ group, T592S or T592E. However, in addition the non-phosphorylatable mutants in the Tet^stable^ group together with T592A, that could all restrict HIV-1 infection in differentiated cells, were also not able to restrict HIV-1 in these cycling cells (Fig. 3B) suggesting that prevention of SAMHD1 phosphorylation and maintenance of a stable tetramer is not enough to restrict HIV-1 replication in cycling cells.

A plausible explanation to these observations is that the steady state dNTP levels in cycling cells, driven by ribonucleotide reductase (RNR) synthesis, is high enough to enable viral replication, regardless of any dNTP hydrolysis by SAMHD1. We have also previously proposed that high dNTP levels could help maintain tetramers of SAMHD1, even when SAMHD1 was phosphorylated [5]. Therefore, to assess if dNTP levels correlated with restriction phenotype of the T592 mutants we measured the dNTP levels in both cycling and differentiated U937 cells expressing our panel of SAMHD1 mutants. Figure 3C, D shows that WT SAMHD1 was able to reduce dNTP levels to ~ 20% of those in the untransduced U937 cells or cells expressing the catalytically dead R164A and HD206-7AA mutants in both differentiated and cycling cells, although the absolute levels in cycling cells always remained much higher than in differentiated cells. This reduction was observed for each individual dNTP measured as well as the sum dNTP pool. Interestingly, all the T592 mutants were also able to reduce dNTP levels similarly to WT in both differentiated and cycling cells (Fig. 3D). This indicates that the mutants have functional triphosphohydrolase activity in cells and agrees with our in vitro findings that most mutants are catalytic active (Additional file 1: Figure S4, Table 1). Strikingly however, tetramer stability and restriction activity did not correlate with dNTP levels in either cell state.

### RNR inhibition does not enable Tet^stable^ mutants to restrict HIV-1 in cycling cells

Our measurements showed that cycling cells expressing T592 mutants had higher levels of dNTPs than the differentiated cells expressing the equivalent mutant (Fig. 3C, D). In addition, dNTP levels in cycling cells were approximately equivalent to those in differentiated U937 cells not expressing SAMHD1 or those expressing catalytically dead SAMHD1 mutants (25 pmol/10^6^ cells). We therefore postulated that HIV-1 restriction might manifest for the stable mutants in cycling cells if the dNTP levels were lowered to those found in differentiated cells. To test this, we treated cycling cells with the RNR inhibitor, hydroxy urea (HU) [33, 45–48]. Since RNR catalyses the rate limiting step of dNTP synthesis in the cell, knock-down should decrease the dNTP pool. We first determined that the dNTP pool in cycling U937 cells could be reduced to approximately that in differentiated cells after 72 h treatment with 1 mM HU (Fig. 3E, orange line). Expressing SAMHD1 in cycling U937 cells reduced dNTPs to a similar level as HU treatment (Fig. 3E, black line) and combined SAMHD1 expression and HU treatment in cycling cells was able to further reduce dNTP levels to those observed in differentiated cells expressing SAMHD1 (Fig. 3E, blue line). Subsequently we tested restriction in cycling cells transduced with WT SAMHD1, the Tet^stable^ mutants T592V and T592C, and the controls HD206-7AA and R164A, with and without HU treatment. Surprisingly, even under these conditions, neither WT SAMHD1 nor mutants T592V and T592C restricted HIV-1 (Fig. 3F), although HU treatment did slightly reduce overall infectivity by enhancing cell toxicity (Additional file 1: Figure S5A). This suggests that, despite lowering the absolute dNTP levels, a stable, catalytically competent SAMHD1 tetramer is still not sufficient to restrict HIV-1 in cycling cells. Therefore, although the stability of SAMHD1 tetramers influences restriction in differentiated cells, this is not observed in cycling cells.

### SAMHD1 can restrict HIV-1 in cycling cells if reverse transcriptase is attenuated

**Fig. 4:**
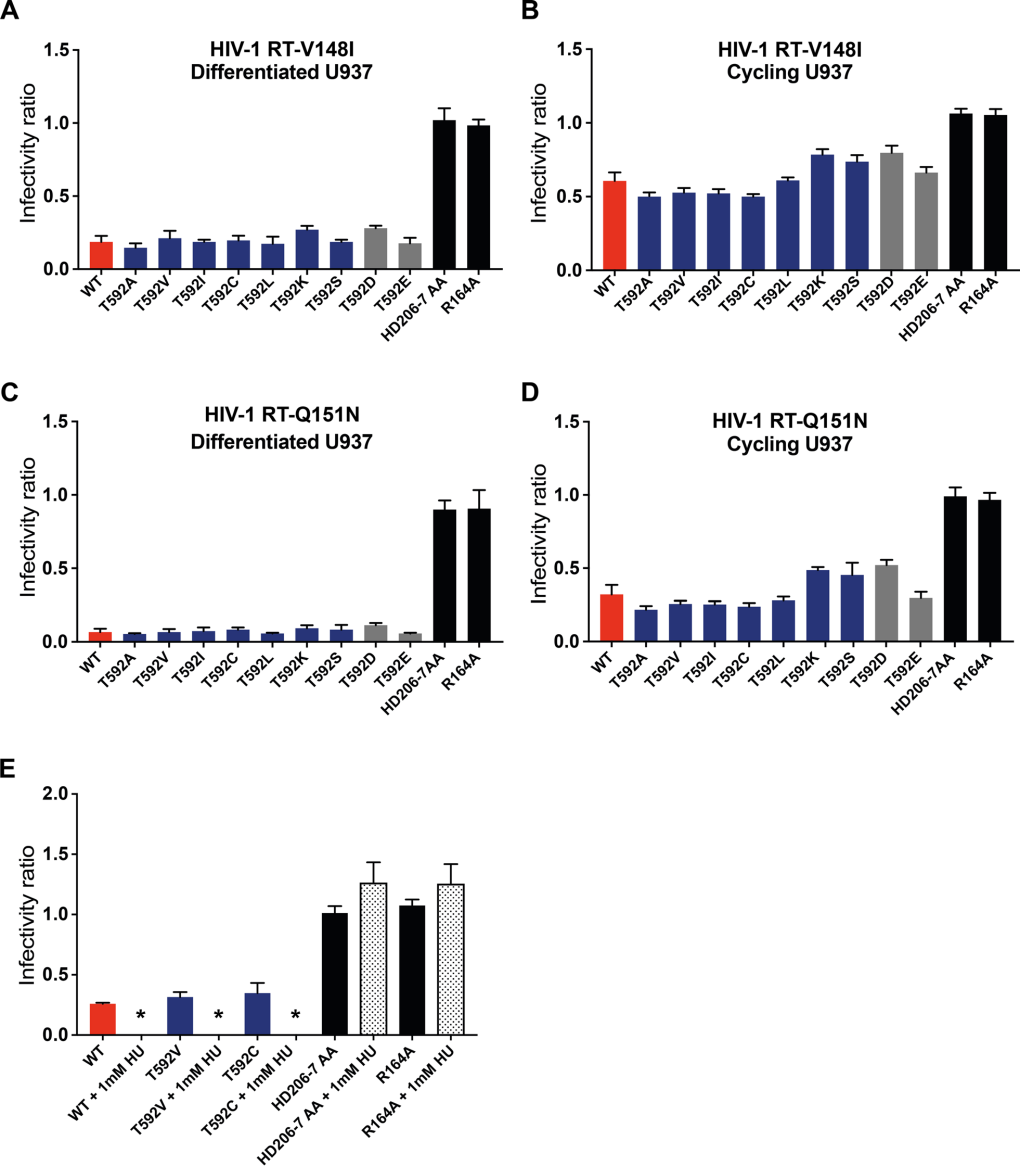
SAMHD1 restriction in U937 cells infected with HIV-1 RT V148I and Q151N mutants Restriction of **A** and **B** HIV-1 RT-V148I and **C** and **D** RT-Q151N RT VLPs in **A** and **C** differentiated and **B** and** D** cycling U937 cells expressing the indicated SAMHD1 T592 mutant. Cells were first transduced with a bicistronic construct expressing WT SAMHD1 or the indicated mutant and YFP, followed by infection with HIV-GFP. Cells were harvested after 72 h and analysed by flow cytometry. The infectivity ratio was calculated by dividing the percentage of GFP positive cells in the YFP (SAMHD1) positive population by the percentage of GFP positive cells in the YFP (SAMHD1) negative population. The bars show the average of three biological repeats and error bars represent the SD. The T592 mutants, phosphomimetic mutants, and inactive negative controls are shown in blue, grey, and black, respectively. **E** Restriction assay of HIV-1 RT-Q151N in cycling U937 cells transduced with WT SAMHD1 (red bars), T592V or T592C mutants (blue bars) or catalytically inactive HD206-7AA and R164A controls (black bars) following 1 mM HU treatment for 72 h (dotted bars) or untreated (solid bars). Restriction assay was performed as above. The bars show the average of at least three biological repeats and error bars represent the SD. *The low abundance of double positive cells (GFP^+^YFP^+^  < 0.05%) for WT SAMHD1, T592V, and T592C HU-treated cells prevented determination of a reliable infectivity ratio

Although we did not find a difference in the dNTPs levels of the bulk population of either cycling or differentiated cells expressing stable or unstable SAMHD mutants, it is possible that our assay is not sensitive enough to detect differences in differentiated cells where the dNTP levels are very low. Additionally, local dNTP concentration and availability and/or cell compartment distribution could be altered in infected cells. Therefore, to look at the effects of dNTP concentration on restriction, we took an orthogonal approach, by using HIV-1 Reverse Transcriptase (RT) mutants, V148I and Q151N, that impair RT activity and that we have previously shown increase HIV-1 sensitivity to SAMHD1 restriction [5]. The V148I and Q151N mutants were chosen as they decrease the dNTP- affinity by 26- and 234-fold, increasing the *K*_*D*_ from the WT value of 1.1 µM to 28.5 µM and 257.6 µM respectively, whilst maintaining WT catalytic activity (*k*_*pol*_) [49, 50]. By comparison, the reported SAMHD1 *K*_*m*_ values for deoxynucleotide substrates range from 10^–4^ to 10^–5^ M [23, 49–54]. Therefore, whilst WT RT has a greater affinity for dNTPs than SAMHD1 the RT mutants have either comparable (V148I) or weaker (Q151N) affinity with respect to the SAMHD1 *K*_*M*_. As expected, WT SAMHD1 was able to restrict HIV-1 RT-V148I (Fig. 4A) and HIV-1 RT-Q151N (Fig. 4C) in differentiated U937 cells, with restriction increasing as the RT dNTP affinity decreased. Interestingly, both Tet^stable^ and Tet^unstable^ mutants also inhibited replication of these viruses (Fig. 4A, C). Moreover, for the first time, we were able to observe restriction of HIV-1 in cycling U937 cells (Fig. 4B, D). Again, all the T592 mutants displayed some capacity to restrict viral replication compared to the negative controls with the Tet^stable^ mutants generally showing the strongest level of restriction. Importantly, restriction of the HIV-1 RT mutants now correlated with total dNTP levels measured in Fig. 3, restriction being stronger in differentiated cells than cycling cells that had higher dNTP levels. In support of this observation, when HU was added to the cycling cells during infection, replication was completely abolished in cells expressing catalytically active WT SAMHD1 and Tet^stable^ mutants (Fig. 4E and Additional file 1: Figure S5B). In addition, we also observed similar, but less pronounced, restriction of the HIV-1 RT mutants by the panel of T592 SAMHD1 mutants in a cycling Jurkat T-cell line (Additional file 1: Figure S6).

Taken together, these results demonstrate that SAMHD1 restriction is highly dependent on the affinity of the viral RT for dNTPs, implying that the mechanism of SAMHD1 restriction does indeed involve lowering dNTP levels. Importantly, we have shown that WT SAMHD1 is not just able to restrict infection of differentiated cells but can restrict HIV-1 in cycling cells when viral replication is sensitised to dNTP levels.

### SAMHD1 expression levels correlate with restriction

**Fig. 5:**
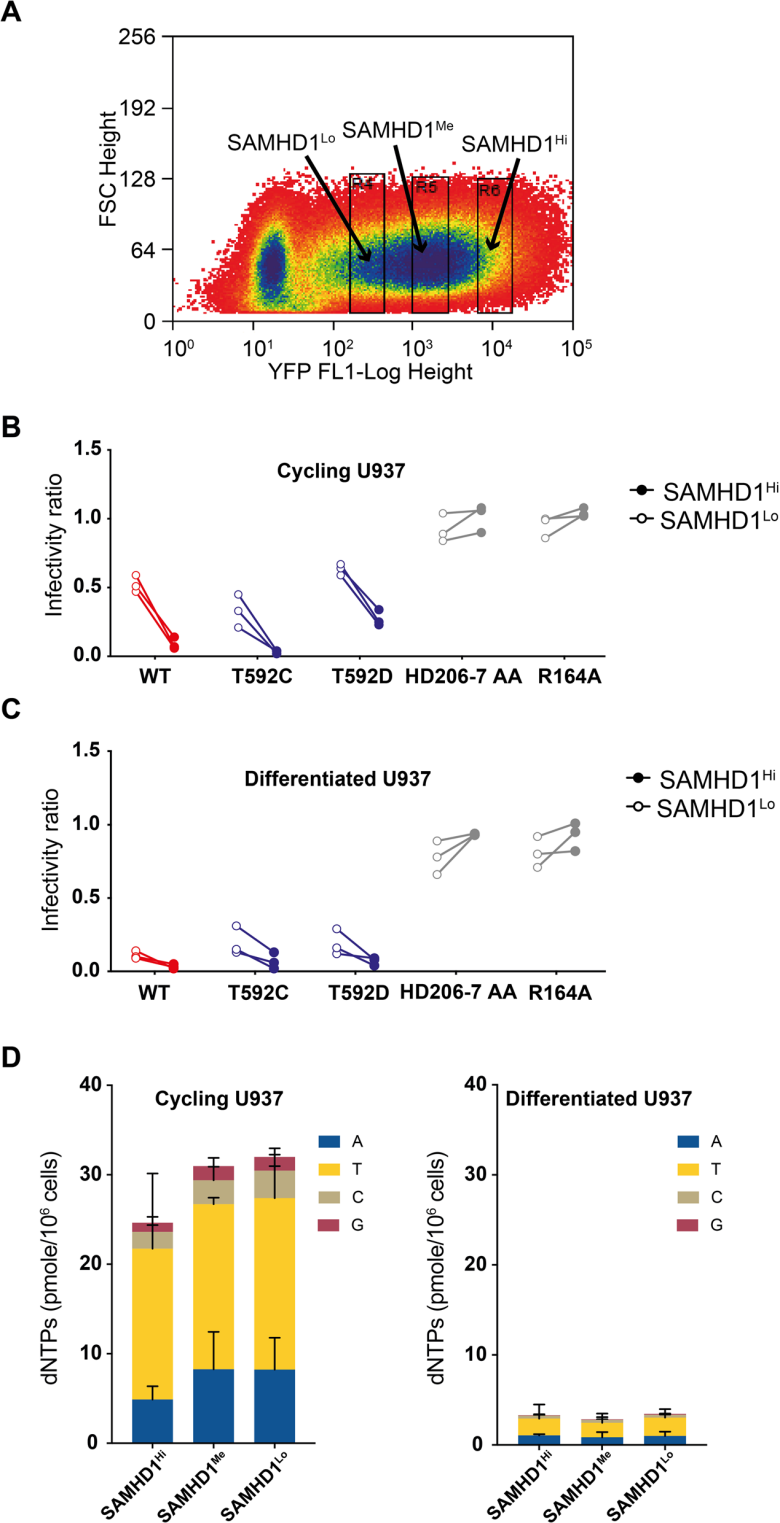
The effect of high and low SAMHD1 expression levels. **A** Plot of flow cytometry data indicating the populations of WT-SAMHD1 transduced U937 cells sorted as high (SAMHD1^Hi^), medium (SAMHD1^Me^) and low (SAMHD1^Lo^) expressing populations. **B**–**C** Restriction of HIV-1-GFP RT-Q151N by SAMHD1^Hi^ (solid circles) and SAMHD1^Low^ (open circles) cells expressing the indicated SAMHD1 proteins (WT, T592C, T592D, HD206-7AA and R164A) in **B** cycling and **C** differentiated U937 cells. The infectivity ratio was calculated as the percentage of GFP positive cells in the YFP (SAMHD1) positive population divided by the percentage of GFP positive cells in the YFP (SAMHD1) negative population. **D** Cell dNTP levels measured from 10^6^ sorted cycling (left) and differentiated (right) U937 cells with SAMHD1^Hi^, SAMHD1^Me^, and SAMHD1^Lo^ expression. The amounts of dATP, TTP, dCTP, and dGTP within the total bar stack are shown in blue, yellow, beige, and red respectively. The values are the mean of two technical repeats and error bars represent the SD

Having observed restriction in cycling cells, we wanted to confirm that the restriction of the HIV-1 RT mutants was due to SAMHD1 activity. We therefore examined the effect of varying the SAMHD1 expression levels on restriction. First, SAMHD1-expressing cells were sorted by flow cytometry and gated into SAMHD1^Hi^, SAMHD1^Me^ and SAMHD1^Lo^ populations based on the fluorescence intensity of YFP expressed from the IRES in the SAMHD1 mRNA (Fig. 5A). In both cycling and differentiated U937 cells transduced with either WT SAMHD1, the Tet^stable^ T592C mutant or the Tet^unstable^ T592D mutant, restriction of HIV-1 RT Q151N was greater in the SAMHD1^Hi^ population than the SAMHD1^Lo^ population. In cells expressing the inactive mutants, HD206-7AA or R164A, HIV-1 RT Q151N was equally or more infectious in the SAMHD^Hi^ population (Fig. 5B, C). The effect was stronger in the cycling cells and, correspondingly, dNTP levels were slightly lower in the SAMHD1^Hi^ population of cycling cells than the SAMHD1^Lo^ population (Fig. 5D). In the differentiated cells, total dNTP levels were much lower and there was less observable difference in the levels in SAMHD1^Hi^, SAMHD1^Me^ and SAMHD1^Lo^ populations (Fig. 5D).

## The level of reverse transcription depends on the RT dNTP-affinity and SAMHD1 tetramer stability

**Fig. 6:**
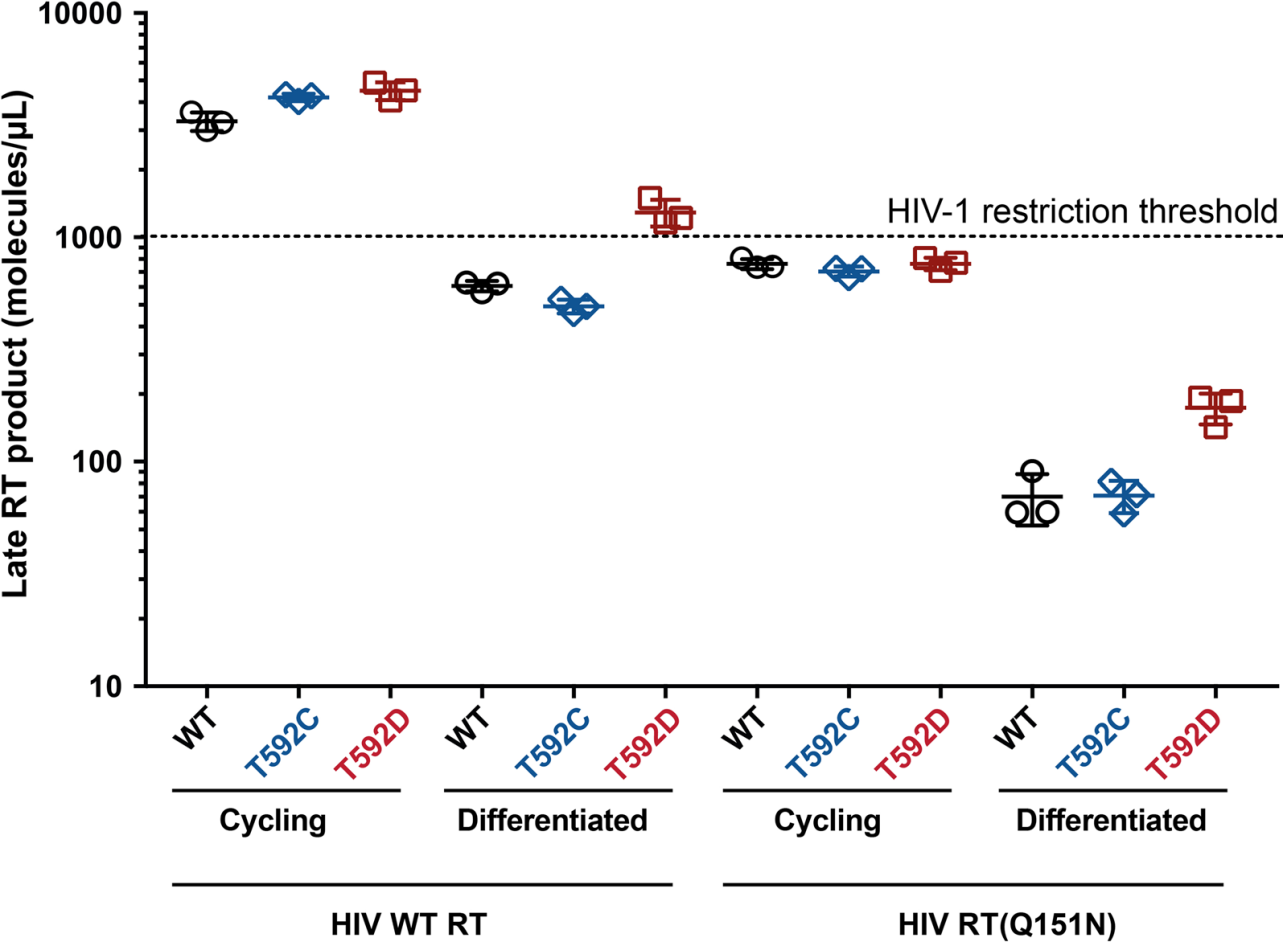
HIV-1 cDNA levels synthesised under different conditions Cycling or differentiated U937 cells transduced with WT SAMHD1 (black circles), T592C (blue diamonds) or T592D (red squares) mutants were infected with WT HIV-1 or RT Q151N mutant VLPs for 48 h. Late viral reverse transcription products were detected by qPCR. Three technical repeats are shown, with the error bars representing SD. The dashed line represents the putative threshold for productive infection over restriction

The restriction assay directly compares replication in the presence and absence of SAMHD1 to calculate an infection ratio whilst not reporting on absolute infection levels. However, given that the RT Q151N mutation might be expected to reduce nascent viral cDNA production, we analysed the effects of SAMHD1 tetramerisation on restriction of WT HIV-1 and the RT Q151N by qPCR quantification of viral cDNA after infection of both cycling and differentiated U937 cells expressing either WT SAMHD1, the Tet^stable^ T592C mutant or the Tet^unstable^ T592D mutant. Additional file 1: Figure S7 shows the amount of early (strong stop) and late (second strand) cDNA products that accumulate for WT HIV-1 and the RT Q151N viruses at time post infection in the presence of WT SAMHD1. As expected, the HIV-1 RT Q151N virus produced less cDNA than WT HIV-1 at all time points, and lower levels of cDNA accumulated in differentiated cells than cycling cells for both viruses. The amount of late cDNA product present at 48 h post infection for all the conditions tested, is plotted in Fig. 6. Inspection of these data shows that SAMHD1 tetramer stability does not affect cDNA levels in cycling cells, regardless of the affinity of the viral RT for dNTPs or the absolute levels of cDNA produced. However, in differentiated cells, there is clearly increased reverse transcription in cells expressing the Tet^unstable^ T592D mutant compared to the Tet^stable^ T592C mutant or WT SAMHD1, again, regardless of the virus used in the infection. Importantly, the amount of cDNA in WT HIV-1 infected, differentiated cells transduced with SAMHD1 T592D is greater than the amount of cDNA produced in cycling cells infected with HIV-1 RT Q151N. The levels of cDNA therefore correlate with restriction, where SAMHD1 T592D is unable to restrict WT HIV in differentiated cells and HIV-1 RT Q151N is restricted by SAMHD1 WT, T592C and T592D in cycling cells (Figs. 3A, 4D). Comparing the conditions that restricted viral infection and those that did not suggests that the threshold for SAMHD1-mediated viral restriction is approximately 1000 molecules of late reverse transcription products (per µL of normalised total cell extract), Fig. 6. Therefore, these findings demonstrate that the stability of SAMHD1 tetramers influences cDNA production most strongly in differentiated cells. The results also corroborate that SAMHD1 restriction is subject to the efficiency of viral reverse transcription in different cells and that differentiation, stabilisation of SAMHD1 tetramers and the affinity of RT for dNTPs all influence the restriction phenotype by impeding reverse transcription.

## Discussion

In this study, we first showed the reversibility of SAMHD1 restriction of HIV-1, by performing a Vpx rescue assay (Fig. 1). This, together with direct in vitro RNA/DNA nuclease assays (Additional file 1: Figure S2), showed that restriction is not due to degradation of the HIV-1 genome as some papers have proposed [36, 37, 39, 40, 55]. Although the involvement of RNA binding cannot be discounted, it seems likely that it is the dNTP triphosphohydrolase activity of SAMHD1 that is responsible for restriction.

SAMHD1 phosphorylation has been likened to an “on/off switch” for triphosphohydrolase activity and it has been hypothesised that this is why phosphomimetic mutants are incapable of restricting HIV-1 and why there is no SAMHD1 restriction of HIV-1 in cycling cells [19, 35, 56]. Previously, we suggested that high dNTP levels found in cycling cells promote tetramerisation and catalytic activity of an unstable phospho-T592 SAMHD1 [5] and proposed this as an explanation for why phosphorylated SAMHD1 was still active in vitro. Although there are many conflicting reports, similar observations were reported by Bhattacharya et al. [34], and more recently by Orris et al. [57], where they found that phosphomimetic mutations affect the kinetics of tetramer assembly and disassembly, without affecting tetramerization equilibrium and overall triphosphohydrolase activity. Here, using our panel of T592 stable and unstable tetramer mutants (Fig. 2), we observed that in cycling cells, neither Tet^stable^ nor Tet^unstable^ T592 SAMHD1 mutants were able to restrict WT HIV-1 (Fig. 3B). Moreover, both Tet^stable^ and Tet^unstable^ mutants result in similar cellular abundance of dNTPs, late reverse transcription products and infectivity (Figs. 3B, D and 6). Excitingly however, using two well-studied point mutations in the HIV-1 RT that lower the dNTP binding affinity and hence the rate of viral cDNA synthesis, we showed for the first time that SAMHD1 can restrict HIV-1 in cycling cells **(**Fig. 4). The strength of the restriction correlated inversely with the efficiency of RT dNTP incorporation rate in both myeloid and lymphocyte cell lines (Fig. 4 and Additional file 1: Figure S6). The fact that restriction was increased in cells with the highest expression of SAMHD1 confirmed that restriction was due to SAMHD1 (Fig. 5**).** Furthermore, adding hydroxyurea in combination with SAMHD1 expression, to reduce dNTP levels further, completely abolished replication of HIV-1 RT-Q151N in cycling U937 cells **(**Fig. 4). This implies that SAMHD1 is competent to restrict HIV-1 replication in cycling cells and that dNTP concentrations are critical for restriction as in differentiated cells. Thus, we do not regard phosphorylation as an on/off switch for SAMHD1 activity and suggest that other factors such as the stage of the cell cycle and overall dNTP levels can affect restriction. Interestingly, whilst all T592 mutants restricted RT-attenuated HIV-1 to some degree, in general the Tet^stable^ group restricted HIV-1 slightly more than the Tet^unstable^ group (Fig. 4B, D). This may reflect subtle differences in dNTP levels in cells expressing different SAMHD1 proteins.

**Fig. 7:**
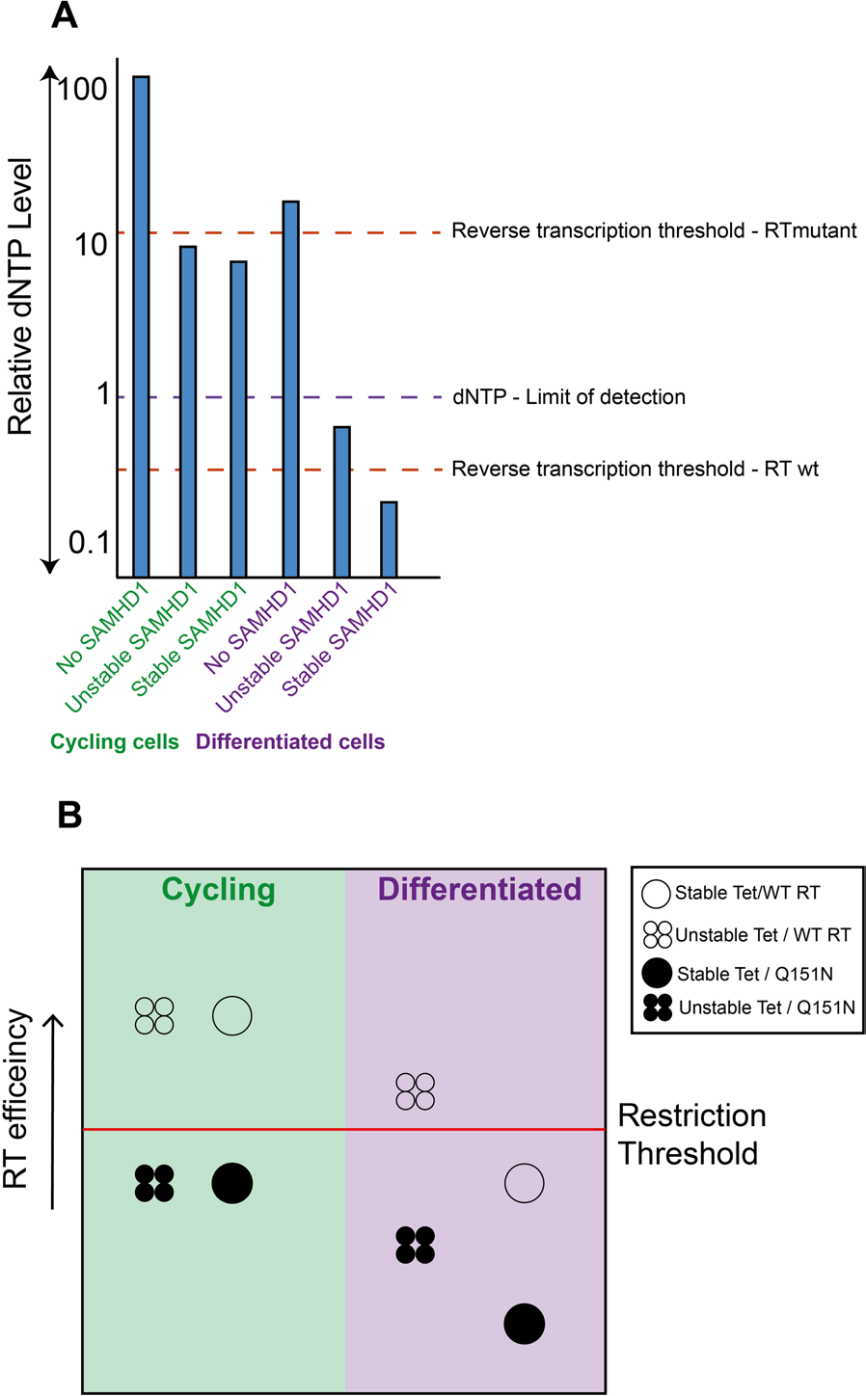
Models of how SAMHD1 variants affect dNTP levels and HIV-1 restriction. **A** A schematic depicting relative dNTP levels in cells expressing different variants of SAMHD1. The graph depicts combined data from our dNTP assays in U937 cells together with projected levels below the assay level of detection. Red lines indicate the thresholds for reverse transcription of WT and Q151N RT mutant HIV-1. Purple line show the limit of detection of the dNTP assay. **B** A schematic model demonstrating the relationship between virus restriction, SAMHD1 stability and HIV-1 RT efficiency. In cycling cells (left, green quadrants), with high basal dNTP levels, SAMHD1 is unable to restrict viral replication of WT HIV-1 (open symbols) with a highly efficient RT, regardless of tetramer stability. By contrast when HIV RT is made less efficient through the introduction of Q151N mutant (black symbols), both stable and unstable tetramer mutant SAMHD1 can now restrict. In differentiated cells (right, purple quadrants), where the dNTP levels are already reduced, WT and stable SAMHD1 tetramer mutants restrict HIV-1, but tetramer destabilising mutants cannot. Restriction is restored against the RT deficient Q151N virus with stable tetramer SAMHD1 reducing replication to even lower levels

This phenotype is mirrored in differentiated cells, where we observed that tetramer stability more strongly impacted restriction (Fig. 3A). The low cellular dNTP levels in differentiated cells are unlikely to support tetramerisation of phospho-T592 or Tet^unstable^ SAMHD1 mutants, which presumably allows dNTP levels to rise enough to support reverse transcription. Unfortunately, we could not detect any differences in overall dNTP levels upon expression of different T592 mutants **(**Fig. 3C**)**, suggesting that our assay is not sensitive enough to detect these variations and that the dNTP levels in differentiated cells expressing SAMHD1 are at/below the level of detection. If it were possible to detect dNTP levels in individual cells, or even at specific cellular sites, then spikes of viral replication in these cells might be observed as the local dNTP levels rise. The increased restriction seen in differentiated cells might also reflect that reverse transcription itself is slower in these cells, likely due to reduced dNTP availability, or that reverse transcription occurs at a different cellular location [58–61]. However, since the *k*_*pol*_ of the HIV-1 RT mutants we have employed remains the same as the WT HIV-1 [18, 49] we prefer the notion that for restriction of HIV-1 RT mutants in cycling cells the local dNTP abundance is the driving factor. By combining all our data, we have attempted to derive a schematic model for how SAMHD1 affects dNTP levels in different scenarios (Fig. 7A) and how different factors influence HIV-1 replication in the presence of SAMHD1 (Fig. 7B).

Interestingly, recent reports have suggested a role for SAMHD1 monomer/dimers in DNA repair [62–64]. Decreased levels of dNTPs at these sites may influence the switch from triphosphohydrolase to single stranded DNA (ssDNA) binding functions, by influencing SAMHD1 tetramerisation, particularly if the SAMHD1 is phosphorylated. Moreover, a more unstable tetramer may promote ssDNA binding over triphosphohydrolase activity. More work is needed to dissect these different functions of SAMHD1 in cycling cells and to relate DNA repair and restriction. SAMHD1 can also be acetylated at residue K405 [65] and SUMOylated at K595 [66]. We have not investigated how substitutions at T592 affect either of these post-translational modifications nor how they influence in vivo triphosphohydrolase activity, localisation, or stability in cells, but these parameters could impact on all functions of SAMHD1.

## Conclusions

Our data suggest a model whereby in an HIV-1 infected cell, if the dNTP level is above a set threshold, then reverse transcription can proceed, resulting in an infection. If this threshold dNTP level is not met, because SAMHD1 is reducing the dNTP pool, then infection will be restricted (Fig. 7). SAMHD1 deoxynucleotide triphosphohydrolase activity reduces dNTP levels in both differentiated and cycling cells but this is offset in cycling cells by RNR activity synthesizing dNTPs. At low dNTP levels, tetramer stability becomes an important determinant of SAMHD1 enzymatic activity and therefore determines how low dNTP levels drop. Wild type HIV-1 is only restricted at very low levels of dNTPs due to the high affinity of HIV-1 reverse transcriptase for dNTPs, which is why restriction is only observed in differentiated cells expressing stable SAMHD1 mutants. This feature of HIV-1 RT also protects this virus from SAMHD1 restriction in cycling cells and is likely why HIV-1 does not carry a Vpx protein, unlike HIV-2. Thus, restriction does depend upon the stability of SAMHD1 tetramers depending on the differentiation state of the cell (and therefore steady state dNTP levels) as well as the affinity of RT for dNTPs.

## Methods

### Plasmids

The SAMHD1-YFP expression plasmid, pLGateway_SAMHD1IRESYFP (template) and phospho-mutants T592A, T592D, and T592E, along with negative controls HD-206,7-AA and R164A (mutations at the catalytic site) are described in our previous study [5]. The remainder of the phospho-mutants were created using either Quikchange II Site-directed Mutagenesis Kit (Agilent Technology) or cloning gene synthesis fragments (GeneArt^®^Strings^™^ DNA Fragment; ThermoFisher) into the template using restriction digestion followed by ligation with SacII and ClaI. The mutagenesis primers and the sequence of synthetic DNA fragments are listed in Additional file 1: Table S1. Plasmids used to make MLV and HIV-1 virus like particles (VLPs): pKB4 (MLV Gag-Pol), p8.91 (HIV-1 Gag-Pol), pVSV-G (envelope protein) and pCSGW (GFP lentiviral vector) have been described previously [5]. The reverse transcriptase (RT) point mutations V148I and Q151N were synthesized by site-directed mutagenesis in p8.91 as previously described [5]. Plasmids used to make SIVmac VLP with (pSIV3^+^) or without Vpx (pSIV3^+^vpx-) have also been described previously [67].

For recombinant expression in *E. coli*, the DNA sequence for WT full-length human SAMHD1 (M1-M626) was inserted by ligation independent cloning into a pET52b( +) expression vector (Novagen) to create an N-terminal StrepII-tag fusion. T592 mutants were generated by site-directed mutagenesis using KOD Hot Start DNA Polymerase (Novagen). DNA primer sequences are listed in Additional file 1: Table S2 mutations were confirmed by DNA sequencing.

### Cells and virus production

293 T cells were maintained in DMEM (Invitrogen) and U937 and Jurkat T-cells were cultured in RPMI containing [L]-Glutamine (ThermoFisher) at 37 °C in 5% CO_2_. Media were supplemented with 10% heat-inactivated foetal calf serum (Biosera) and 1% penicillin/streptomycin (Sigma). MLV-based VLPs used to transduce cells with SAMHD1 were produced by co-transfecting 293 T cells with pVSV-G, pKB4 and pLGateway_SAMHD1IRESYFP (either WT or SAMHD1 mutant). HIV-1-GFP VLPs were produced by co-transfecting 293 T cells with pCSGW, pVSV-G, and p8.91 (either WT or RT mutant). SIVmac VLPs containing or lacking Vpx were produced by co-transfecting 293 T cells with pVSV-G and either pSIV3^+^ (for Vpx +) or pSIV3^+^vpx- (for Vpx-). VLP-containing cell culture supernatants were harvested 48 h post-transfection and titred on TE671 or 293 T cells for normalisation prior to infection. VLPs with mutations in Reverse Transcriptase were normalised by HIV-1 p24 ELISA (Perkin Elmer).

### Vpx rescue of HIV-1 infection assay

Parental U937 cells or stable U937 lines expressing WT or SAMHD1 deletion mutant (1–583) at a density of 1 × 10^5^ cells per well (12-well plate) were differentiated by addition of 100 nM Phorbol-12myristate 13-acetate (PMA, Sigma) for three days. Differentiated cells were then infected with HIV-1-GFP (~ 2 ng CA) in 750 µL RPMI and SIVmac VLPs with (Vpx +) or without (Vpx-) packaged Vpx were added in a final volume of 250 µL to one well each at the following time points: 0, 2, 4, 6, 8, 10, 12, 24 and 48 h post-infection with HIV-GFP. The dilution of SIV VLP used was the minimum dilution required to completely degrade NLS-GFP-SAMHD1(600–626) in a Degron assay as previously described [67, 68]. Cells were harvested at 96 h post-HIV-1-GFP infection and analysed for GFP expression by flow cytometry using the BDVERSE software package. The ratio of GFP positive cells in samples receiving Vpx compared to samples not receiving Vpx was calculated and plotted.

### Nevirapine washout assay

Parental U937 cells (0.5 × 10^5^ per well of a 12-well plate) were differentiated in 100 nM final concentration of PMA (Sigma) for three days. Cells were then treated with RPMI media with or without 10 µM Nevirapine and infected with HIV-GFP. Nevirapine was subsequently removed by washing at time-points between 0 and 48 h. Cells were harvested at 96 h post-infection and analysed for HIV-GFP expression by flow cytometry using the BDVERSE software package.

### Recombinant SAMHD1 expression and purification

Cleavable StrepII-tagged SAMHD1 constructs were expressed in *E. coli* strain Rosetta 2 (DE3) (Novagen) in Terrific Broth media. Cultures were initially grown at 37 °C with shaking. Protein expression was induced by addition of 0.4 mM IPTG to log phase cultures (A_600_ = 0.8) and the cells were incubated for a further 20 h at 16 °C. Cells were harvested by centrifugation and resuspended in 50 mL (per 10 g cell pellet) lysis buffer [50 mM Tris–HCl pH 7.8, 500 mM NaCl, 4 mM MgCl_2_, 0.5 mM TCEP,1 × cOmplete^™^ EDTA-free protease inhibitor cocktail (Roche)]. The cells were lysed by sonication and the lysate was clarified by centrifugation for 1 h at 50,000 ×*g *and 4 °C. The supernatant fraction was applied to a 10 mL StrepTactin-XT (IBA) affinity column, followed by 300 mL of wash buffer (50 mM Tris–HCl pH 7.8, 500 mM NaCl, 4 mM MgCl_2_, 0.5 mM TCEP) at 4 °C. Bound proteins were eluted from the column by circulation of 0.5 mg of GST-tagged PreScission Protease (GE) in 25 mL of wash buffer over the column in a closed circuit overnight. The supernatant (25 mL) and an additional elution of 15 mL wash buffer were pooled and concentrated to 2.5 mL. PreScission Protease was removed by affinity chromatography using a 1 mL GSTrap HP column (GE) and the eluent was applied to a Superdex 200 26/60 (GE) size exclusion column equilibrated with a gel filtration buffer of 10 mM Tris–HCl pH 7.8, 150 mM NaCl, 4 mM MgCl_2_, 0.5 mM TCEP. Peak fractions from the column eluent containing SAMHD1 were concentrated to approximately 20 mg mL^−1^ and flash-frozen in liquid nitrogen in small aliquots.

### SEC–MALLS

Size exclusion chromatography coupled to Multi-Angle Laser Light Scattering (SEC–MALLS) was used to determine the molar mass composition of SAMHD1 samples upon addition of GTP and dATP (ThermoFisher Scientific). For assessment of tetramer stability, 30 μM WT or T592 mutant SAMHD1 was incubated at room temperature for 5 min, 1 h or 3 h after the addition of 0.2 mM GTP and 0.5 mM dATP prior to column loading. Samples (100 μL) were then applied to a Superdex 200 10/300 INCREASE GL column equilibrated in 20 mM Tris–HCl pH 7.8, 150 mM NaCl, 5 mM MgCl_2_, 0.5 mM TCEP and 3 mM NaN_3_ at a flow rate of 1.0 mL min^−1^. The scattered light intensity and protein concentration of the column eluate were recorded using a DAWN HELEOS laser photometer and an OPTILAB-rEX differential refractometer (dRI) (dn/dc = 0.186), respectively. The weight-averaged molecular mass of material contained in chromatographic peaks was determined using the combined data from both detectors in the ASTRA software version 6.1 (Wyatt Technology Corp., Santa Barbara, CA).

### NMR analysis of SAMHD1 catalysis

1D ^1^H NMR spectroscopy was used to measure GTP-stimulated SAMHD1 hydrolysis rates of dATP. Reactions were prepared in NMR buffer (20 mM Tris–HCl pH 8.0, 150 mM NaCl, 5 mM MgCl_2_, 2 mM TCEP, 5% (v/v) D_2_O) containing 0.2 mM GTP, 0.5 mM dATP and 1 μM of WT or T592 mutants. ^1^H NMR spectra (2 dummy scans, 4 scans) were recorded at 30 s intervals as a pseudo 2D array at 293 K using either a Bruker Avance III 600 MHz or Avance IIIHD 700 MHz NMR spectrometer equipped with a 5 mm TCI cryoprobe. Solvent suppression was achieved using excitation sculpting [69]. Experiments were typically carried out for 1 h. The integrals for clearly resolved substrate and product peaks at each time-point were extracted using the Bruker Dynamics Centre software package and used to construct plots of substrate or product against time. Initial rates were extracted from the linear part of the curve in order to determine apparent *k*_cat_ values.

### Nuclease assays

Nucleic acids (10 pmol) were 5′ end-labelled using (10 U) T4 polynucleotide kinase and (20 pmol) γ-^32^P-ATP in 1 × T4 polynucleotide kinase buffer (ΝΕΒ) at 37 °C for 30 min then boiled for 30 s to heat inactive the enzyme. Labelled nucleic acids were purified from unincorporated ATP using Illustra MicroSpin G-25 columns (GE Healthcare) and added to unlabelled to give a final concentration of 0.5 μM. Time courses of nuclease digestion were performed with 30 μM SAMHD1 and 0.5 μM each nucleic acid substrate in a reaction buffer containing 10 mM Tris–HCl pH 7.8, 150 mM NaCl, 10 mM MgCl_2_, 0.5 mM TCEP. Reactions were quenched at between 0 and 90 min by the addition of formamide loading buffer and boiling. Reaction products were resolved using 8 M urea denaturing 20% polyacrylamide gels (19:1 acrylamide:bis-acrylamide). Gels were electrophoresed for approximately two hours at 45–50 W (~ 1000 V). Gels were fixed in 10% acetic acid, 10% methanol and dried under vacuum at 65 °C. Nucleic acid species were imaged either by autoradiography on X-ray film or by phosphor-imaging using a GE STORM 840 Molecular Imager. Reaction products were quantified using ImageQuant software (GE Healthcare).

### Immunoblotting

SAMHD1-YFP–expressing U937 cells (WT SAMHD1 and mutants) were sorted on a MoFlo XDP flow cytometer (Beckman Coulter). After culturing for 3 to 4 days to recover from cell sorting, 1 × 10^7^ cells were harvested, washed in PBS and lysed by addition of 100 μL of radioimmunoprecipitation assay buffer (ThermoFisher) containing protease inhibitors (Roche), DNase-I (Invitrogen), and phosphatase inhibitor (ThermoFisher) at 4 °C for 1 h. Cell lysates were separated by SDS PAGE and analysed for SAMHD1 and phosphorylated SAMHD1 expression by immunoblotting with mouse anti-SAMHD1 mAb (IF6; GeneTex) 1/2000 and rabbit anti-phospho-SAMHD1 mAb (Cell Signalling Technology) 1/1000, followed by anti-mouse and donkey anti-rabbit IRDye^®^ 800 CW antibody (LI-COR) 1/10,000, respectively. Bands were visualised on an Odyssey Infrared Imager (LI-COR).

### Viral restriction assay

Two-colour flow cytometry restriction assay was carried out as previously described [5, 70]. Briefly, U937 cells (4 × 10^5^ per well in a 12-well plate) were transduced with SAMHD1-YFP (WT or phospho-mutants) to give approximately 30% SAMHD1 positive cells on day 3 post-transduction. After 72 h, cells were split 1/4 into two new 12-well plates – one for cycling cells and the other for differentiated cells. Media or media containing 100 nM PMA was added to the plates respectively for 96 h. The cells were subsequently infected with HIV-1-GFP (to give ~ 30% infection) for a further 72 h, maintaining PMA in differentiated cells). Cells were harvested and analysed for GFP expression by flow cytometry (BD LSRFortessa™). An infectivity ratio was calculated by dividing the percentage of transduced (YFP + ve) cells that were infected (GFP + ve) by the percentage of non-transduced cells that were infected.

### Measurement of deoxynucleoside triphosphate (dNTP) levels in cells

Levels of A, C, G and T deoxynucleotide-triphosphates in cells were measured as previously described [5, 71]. Briefly, U937 cells transduced with SAMHD1-YFP WT and mutants, were sorted for YFP positive cells on a MoFlo^™^ XDP cell sorter (Beckman) and the cell lysates from 2 × 10^5^ cycling or 4 × 10^6^ differentiated cells were analysed for each dNTP. Levels were quantified by radiolabel incorporation assays, performed using the oligonucleotide templates detailed in [72] and the procedures described in [73] with the following modifications: Standard curves ranged from 0.05 to 4 pmol, 1 unit of Taq DNA polymerase (Invitrogen) was used in place of Thermo-Sequenase (GE Healthcare) and 2.5 μM of α-^32^P-dGTP (to measure dATP, dCTP, and TTP) and α-^32^P-TTP (to measure dGTP) were employed as an incorporation label. The result was standardized as the pmole amount of each dNTP per 10^6^ cells.

### Quantification of reverse transcription products

U937 cells transduced with SAMHD1-YFP (WT, T592C or T592D) were sorted for YFP using a MoFlo^™^ XDP cell sorter (Beckman) and differentiated as described above. Both cycling and differentiated cells were plated 4 × 10^5^ cells per well of a 12-well plate and spinoculated with RQ1 DNase (Promega)-treated HIV-1-GFP VLPs (15 ng p24, WT or RT 151N) for 30 min at 16 °C followed by incubation at 37 °C. Cells were harvested at 24-, 48-, 60-, and 72-h post-infection. Cells were washed with ice-cold PBS and trypsinised if differentiated. Cell pellets were flash-frozen on dry ice. Cellular DNA was isolated using the DNeasy Blood & Tissue Kit (Qiagen), followed by DpnI treatment for 2.5 h. The quantitation of strong stop and second strand PCR products was determined by qPCR. PCR reactions contained 9 μM forward and reverse primers (forward primer: TAACTAGGGAACCCACTGC; reverse primer strong stop: GCTAGAGATTTTCCACACTG; reverse primer second strand: CTGCGTCGAGAGAGCTCCTCTGGTT), 2.5 μM probe (FAM- ACACAACAGACGGGCACACACTA-TAMRA), 1X Taqman Fast Advanced Mastermix, and 2 µL DpnI-treated DNA. Reactions were carried out in a 7500 Fast Real-Time PCR System (Applied Biosystems) using standard reaction conditions.

### Data analysis

All analyses were performed in Prism (v7.0c, Graphpad). For statistical analysis, a Mann–Whitney U-test (nonparametric) was performed to compare cells transduced with SAMHD1 and negative controls.

## Supplementary Information

**Additional file 1: Table S1**. T592 mutagenic primers for mammalian expression. **Table S2**. T592 mutagenic primers for E. coli expression. **Figure S1**. Vpx rescue assay outline. **Figure S2**. Testing SAMHD1 Nuclease activity.** Figure S3**. SAMHD1 T592 phospho-mutant panel. **Figure S4**. 1H NMR analysis of dNTP hydrolysis by SAMHD1 T592 mutants. **Figure S5**. Infectivity data for WT HIV and Q151N RT mutant infections in the presence of HU. **Figure S6**. SAMHD1 restriction in Jurkat cells infected with HIV-1 WT, RT V148I and Q151N mutants. **Figure S7**. Accumulation of viral reverse transcripts with time in U937 cells.

## Data Availability

All data generated or analysed during this study are included in this published article and its Additional information files.

## References

[1] Goldstone DC, Ennis-Adeniran V, Hedden JJ, Groom HCT, Rice GI, Christodoulou E, Walker PA, Kelly G, Haire LF, Yap MW (2011). HIV-1 restriction factor SAMHD1 is a deoxynucleoside triphosphate triphosphohydrolase. Nature.

[2] Brandariz-Nuñez A, Valle-Casuso JC, White TE, Laguette N, Benkirane M, Brojatsch J, Diaz-Griffero F (2012). Role of SAMHD1 nuclear localization in restriction of HIV-1 and SIVmac. Retrovirology.

[3] Kim C (2003). SAM domains: uniform structure, diversity of function. Trends Biochem Sci.

[4] Qiao F, Bowie JU (2005). The many faces of SAM. Sci STKE.

[5] Arnold LH, Groom HCT, Kunzelmann S, Schwefel D, Caswell SJ, Ordonez P, Mann MC, Rueschenbaum S, Goldstone DC, Pennell S (2015). Phospho-dependent regulation of SAMHD1 oligomerisation couples catalysis and restriction. PLoS Pathog.

[6] Franzolin E, Pontarin G, Rampazzo C, Miazzi C, Ferraro P, Palumbo E, Reichard P, Bianchi V (2013). The deoxynucleotide triphosphohydrolase SAMHD1 is a major regulator of DNA precursor pools in mammalian cells. Proc Natl Acad Sci USA.

[7] White TE, Brandariz-Nuñez A, Valle-Casuso JC, Amie S, Nguyen L, Kim B, Brojatsch J, Diaz-Griffero F (2013). Contribution of SAM and HD domains to retroviral restriction mediated by human SAMHD1. Virology.

[8] Powell RD, Holland PJ, Hollis T, Perrino FW (2011). aicardi-goutières syndrome gene and HIV-1 restriction factor SAMHD1 Is a dGTP-regulated deoxynucleotide triphosphohydrolase. J Biol Chem.

[9] Bonifati S, Daly MB, St Gelais C, Kim SH, Hollenbaugh JA, Shepard C, Kennedy EM, Kim D-H, Schinazi RF, Kim B, Wu L (2016). SAMHD1 controls cell cycle status, apoptosis and HIV-1 infection in monocytic THP-1 cells. Virology.

[10] Baldauf H-M, Pan X, Erikson E, Schmidt S, Daddacha W, Burggraf M, Schenkova K, Ambiel I, Wabnitz G, Gramberg T (2012). SAMHD1 restricts HIV-1 infection in resting CD4(+) T cells. Nat Med.

[11] Laguette N, Sobhian B, Casartelli N, Ringeard M, Chable-Bessia C, Ségéral E, Yatim A, Emiliani S, Schwartz O, Benkirane M (2011). SAMHD1 is the dendritic- and myeloid-cell-specific HIV-1 restriction factor counteracted by Vpx. Nature.

[12] Berger A, Sommer AF, Zwarg J, Hamdorf M, Welzel K, Esly N, Panitz S, Reuter A, Ramos I, Jatiani A (2011). SAMHD1-deficient CD14+ cells from individuals with aicardi-goutieres syndrome are highly susceptible to HIV-1 infection. PLoS Pathog.

[13] Descours B, Cribier A, Chable-Bessia C, Ayinde D, Rice G, Crow Y, Yatim A, Schwartz O, Laguette N, Benkirane M (2012). SAMHD1 restricts HIV-1 reverse transcription in quiescent CD4+ T-cells. Retrovirology.

[14] Hrecka K, Hao C, Gierszewska M, Swanson SK, Kesik-Brodacka M, Srivastava S, Florens L, Washburn MP, Skowronski J (2011). Vpx relieves inhibition of HIV-1 infection of macrophages mediated by the SAMHD1 protein. Nature.

[15] Ahn J, Hao C, Yan J, DeLucia M, Mehrens J, Wang C, Gronenborn AM, Skowronski J (2012). HIV/simian immunodeficiency virus (SIV) accessory virulence factor Vpx loads the host cell restriction factor SAMHD1 onto the E3 ubiquitin ligase complex CRL4DCAF1. J Biol Chem.

[16] Lim ES, Fregoso OI, McCoy CO, Matsen FA, Malik HS, Emerman M (2012). The ability of primate lentiviruses to degrade the monocyte restriction factor SAMHD1 preceded the birth of the viral accessory protein Vpx. Cell Host Microbe.

[17] Kim B, Nguyen LA, Daddacha W, Hollenbaugh JA (2012). Tight interplay among SAMHD1 protein level, cellular dNTP levels, and HIV-1 proviral DNA synthesis kinetics in human primary monocyte-derived macrophages. J Biol Chem.

[18] Lahouassa H, Daddacha W, Hofmann H, Ayinde D, Logue EC, Dragin L, Bloch N, Maudet C, Bertrand M, Gramberg T (2012). SAMHD1 restricts the replication of human immunodeficiency virus type 1 by depleting the intracellular pool of deoxynucleoside triphosphates. Nat Immunol.

[19] Cribier A, Descours B, Valadão ALC, Laguette N, Luiza C, Laguette N, Benkirane M (2013). Phosphorylation of SAMHD1 by Cyclin A2/CDK1 regulates its restriction activity toward HIV-1. Cell Rep.

[20] Ji X, Wu Y, Yan J, Mehrens J, Yang H, DeLucia M, Hao C, Gronenborn AM, Skowronski J, Ahn J, Xiong Y (2013). Mechanism of allosteric activation of SAMHD1 by dGTP. Nat Struct Mol Biol.

[21] Yan J, Kaur S, DeLucia M, Hao C, Mehrens J, Wang C, Golczak M, Palczewski K, Gronenborn AM, Ahn J, Skowronski J (2013). Tetramerization of SAMHD1 is required for biological activity and inhibition of HIV infection. J Biol Chem.

[22] Zhu C, Gao W, Zhao K, Qin X, Zhang Y, Peng X, Zhang L, Dong Y, Zhang W, Li P, et al. Structural insight into dGTP-dependent activation of tetrameric SAMHD1 deoxynucleoside triphosphate triphosphohydrolase. Worldwide Protein Data Bank. 2013. 10.1038/ncomms3722 24217394

[23] Morris ER, Caswell SJ, Kunzelmann S, Arnold LH, Purkiss AG, Kelly G, Taylor IA (2020). Crystal structures of SAMHD1 inhibitor complexes reveal the mechanism of water-mediated dNTP hydrolysis. Nat Commun.

[24] Tang C, Ji X, Wu L, Xiong Y (2015). Impaired dNTPase activity of SAMHD1 by phosphomimetic mutation of Thr-592. J Biol Chem.

[25] Yan J, Hao C, DeLucia M, Swanson S, Florens L, Washburn MP, Ahn J, Skowronski J (2015). CyclinA2-cyclin-dependent kinase regulates SAMHD1 protein phosphohydrolase domain. J Biol Chem.

[26] Morris ER, Taylor IA (2019). The missing link: allostery and catalysis in the anti-viral protein SAMHD1. Biochem Soc Trans.

[27] Pauls E, Ruiz A, Badia R, Permanyer M, Gubern A, Riveira-Muñoz E, Torres-Torronteras J, Álvarez M, Mothe B, Brander C (2014). Cell cycle control and HIV-1 susceptibility are linked by CDK6-dependent CDK2 phosphorylation of samhd1 in myeloid and lymphoid cells. J Immunol.

[28] St Gelais C, de Silva S, Hach JC, White TE, Diaz-Griffero F, Yount JS, Wu L (2014). Identification of cellular proteins interacting with the retroviral restriction factor SAMHD1. J Virol.

[29] Schott K, Fuchs NV, Derua R, Mahboubi B, Schnellbächer E, Seifried J, Tondera C, Schmitz H, Shepard C, Brandariz-Nuñez A (2018). Dephosphorylation of the HIV-1 restriction factor SAMHD1 is mediated by PP2A-B55α holoenzymes during mitotic exit. Nat Commun.

[30] Mauney CH, Hollis T (2018). SAMHD1: recurring roles in cell cycle, viral restriction, cancer, and innate immunity. Autoimmunity.

[31] Schmidt S, Schenkova K, Adam T, Erikson E, Lehmann-Koch J, Sertel S, Verhasselt B, Fackler OT, Lasitschka F, Keppler OT (2015). SAMHD1's protein expression profile in humans. J Leukoc Biol.

[32] Welbourn S, Dutta SM, Semmes OJ, Strebel K (2013). Restriction of virus infection but not catalytic dNTPase activity is regulated by phosphorylation of SAMHD1. J Virol.

[33] Welbourn S, Strebel K (2016). Low dNTP levels are necessary but may not be sufficient for lentiviral restriction by SAMHD1. Virology.

[34] Bhattacharya A, Wang Z, White T, Buffone C, Nguyen LA, Shepard CN, Kim B, Demeler B, Diaz-Griffero F, Ivanov DN (2016). Effects of T592 phosphomimetic mutations on tetramer stability and dNTPase activity of SAMHD1 can not explain the retroviral restriction defect. Sci Rep.

[35] Herrmann A, Wittmann S, Thomas D, Shepard CN, Kim B, Ferreirós N, Gramberg T (2018). The SAMHD1-mediated block of LINE-1 retroelements is regulated by phosphorylation. Mob DNA.

[36] Beloglazova N, Flick R, Tchigvintsev A, Brown G, Popovic A, Nocek B, Yakunin AF (2013). Nuclease activity of the human SAMHD1 protein implicated in the aicardi-goutieres syndrome and HIV-1 restriction. J Biol Chem.

[37] Choi J, Ryoo J, Oh C, Hwang S, Ahn K (2015). SAMHD1 specifically restricts retroviruses through its RNase activity. Retrovirology.

[38] Goncalves A, Karayel E, Rice GI, Bennett KL, Crow YJ, Superti-Furga G, Bürckstümmer T (2012). SAMHD1 is a nucleic-acid binding protein that is mislocalized due to aicardi-goutières syndrome-associated mutations. Hum Mutat.

[39] Ryoo J, Choi J, Oh C, Kim S, Seo M, Kim S-Y, Seo D, Kim J, White TE, Brandariz-Nuñez A (2014). The ribonuclease activity of SAMHD1 is required for HIV-1 restriction. Nat Med.

[40] Ryoo J, Hwang S-Y, Choi J, Oh C, Ahn K (2016). SAMHD1, the Aicardi-Goutières syndrome gene and retroviral restriction factor, is a phosphorolytic ribonuclease rather than a hydrolytic ribonuclease. Biochem Biophys Res Commun.

[41] Seamon KJ, Bumpus NN, Stivers JT (2016). Single-stranded nucleic acids bind to the tetramer interface of SAMHD1 and prevent formation of the catalytic homotetramer. Biochemistry.

[42] Seamon KJ, Sun Z, Shlyakhtenko LS, Lyubchenko YL, Stivers JT (2015). SAMHD1 is a single-stranded nucleic acid binding protein with no active site-associated nuclease activity. Nucleic Acids Res.

[43] Tüngler V, Staroske W, Kind B, Dobrick M, Kretschmer S, Schmidt F, Krug C, Lorenz M, Chara O, Schwille P, Lee-Kirsch MA (2013). Single-stranded nucleic acids promote SAMHD1 complex formation. J Mol Med.

[44] Maharana S, Kretschmer S, Hunger S, Yan X, Kuster D, Traikov S, Zillinger T, Gentzel M, Elangovan S, Dasgupta P (2022). SAMHD1 controls innate immunity by regulating condensation of immunogenic self RNA. Mol Cell.

[45] Akerblom L, Ehrenberg A, Gräslund A, Lankinen H, Reichard P, Thelander L (1981). Overproduction of the free radical of ribonucleotide reductase in hydroxyurea-resistant mouse fibroblast 3T6 cells. Proc Natl Acad Sci.

[46] Krakoff IH, Brown NC, Reichard P (1968). Inhibition of ribonucleoside diphosphate reductase by hydroxyurea. Can Res.

[47] Lassmann G, Thelander L, Gräslund A (1992). EPR stopped-flow studies of the reaction of the tyrosyl radical of protein R2 from ribonucleotide reductase with hydroxyurea. Biochem Biophys Res Commun.

[48] Nyholm S, Thelander L, Gräslund A (1993). Reduction and loss of the iron center in the reaction of the small subunit of mouse ribonucleotide reductase with hydroxyurea. Biochemistry.

[49] Diamond TL, Roshal M, Jamburuthugoda VK, Reynolds HM, Merriam AR, Lee KY, Balakrishnan M, Bambara RA, Planelles V, Dewhurst S, Kim B (2004). Macrophage tropism of HIV-1 depends on efficient cellular dNTP utilization by reverse transcriptase. J Biol Chem.

[50] Weiss KK, Bambara RA, Kim B (2002). Mechanistic role of residue Gln151 in error prone DNA synthesis by human immunodeficiency virus type 1 (HIV-1) reverse transcriptase (RT): pre-steady state kinetic study of the Q151N HIV-1 RT mutant with increased fidelity. J Biol Chem.

[51] Arnold LH, Kunzelmann S, Webb MR, Taylor IA (2015). A continuous enzyme-coupled assay for triphosphohydrolase activity of HIV-1 restriction factor SAMHD1. Antimicrob Agents Chemother.

[52] Jang S, Zhou X, Ahn J (2016). Substrate specificity of SAMHD1 triphosphohydrolase activity is controlled by deoxyribonucleoside triphosphates and phosphorylation at Thr592. Biochemistry.

[53] Knecht KM, Buzovetsky O, Schneider C, Thomas D, Srikanth V, Kaderali L, Tofoleanu F, Reiss K, Ferreiros N, Geisslinger G (2018). The structural basis for cancer drug interactions with the catalytic and allosteric sites of SAMHD1. Proc Natl Acad Sci USA.

[54] Morris ER, Kunzelmann S, Caswell SJ, Purkiss AG, Kelly G, Taylor IA (2021). Probing the catalytic mechanism and inhibition of SAMHD1 using the differential properties of R(p)- and S(p)-dNTPalphaS diastereomers. Biochemistry.

[55] Brandariz-Nuñez A, Valle-Casuso JC, White TE, Nguyen L, Bhattacharya A, Wang Z, Demeler B, Amie S, Knowlton C, Kim B (2013). Contribution of oligomerization to the anti-HIV-1 properties of SAMHD1. Retrovirology.

[56] St C, Gelais SH, Kim VV, Maksimova OB, Knecht KM, Shepard C, Kim B, Xiong Y, Li W (2018). A cyclin-binding motif in human SAMHD1 is required for its HIV-1 restriction, dNTPase activity, tetramer formation, and efficient phosphorylation. J Virol.

[57] Orris B, Huynh KW, Ammirati M, Han S, Bolaños B, Carmody J, Petroski MD, Bosbach B, Shields DJ, Stivers JT (2022). Phosphorylation of SAMHD1 Thr592 increases C-terminal domain dynamics, tetramer dissociation and ssDNA binding kinetics. Nucl Acids Res.

[58] Collin M, Gordon S (1994). The kinetics of human immunodeficiency virus reverse transcription are slower in primary human macrophages than in a lymphoid cell line. Virology.

[59] Burdick RC, Li C, Munshi M, Rawson JM, Nagashima K, Hu W-S, Pathak VK (2020). HIV-1 uncoats in the nucleus near sites of integration. Proc Natl Acad Sci.

[60] Cosnefroy O, Murray PJ, Bishop KN (2016). HIV-1 capsid uncoating initiates after the first strand transfer of reverse transcription. Retrovirology.

[61] Galvis AE, Fisher HE, Nitta T, Fan H, Camerini D (2014). Impairment of HIV-1 cDNA synthesis by DBR1 knockdown. J Virol.

[62] Wing PA, Davenne T, Wettengel J, Lai AG, Zhuang X, Chakraborty A, D’Arienzo V, Kramer C, Ko C, Harris JM (2019). A dual role for SAMHD1 in regulating HBV cccDNA and RT-dependent particle genesis. Life Sci Alliance.

[63] Daddacha W, Koyen AE, Bastien AJ, Head PE, Dhere VR, Nabeta GN, Connolly EC, Werner E, Madden MZ, Daly MB (2017). SAMHD1 promotes DNA end resection to facilitate DNA repair by homologous recombination. Cell Rep.

[64] Coquel F, Silva M-J, Técher H, Zadorozhny K, Sharma S, Nieminuszczy J, Mettling C, Dardillac E, Barthe A, Schmitz A-L (2018). SAMHD1 acts at stalled replication forks to prevent interferon induction. Nature.

[65] Lee EJ, Seo JH, Park J-H, Vo TTL, An S, Bae S-J, Le H, Lee HS, Wee H-J, Lee D (2017). SAMHD1 acetylation enhances its deoxynucleotide triphosphohydrolase activity and promotes cancer cell proliferation. Oncotarget.

[66] Martinat C, Cormier A, Tobaly-Tapiero J, Palmic N, Casartelli N, Mahboubi B, Coggins SAA, Buchrieser J, Persaud M, Diaz-Griffero F (2021). SUMOylation of SAMHD1 at Lysine 595 is required for HIV-1 restriction in non-cycling cells. Nat Commun.

[67] Schwefel D, Groom HC, Boucherit VC, Christodoulou E, Walker PA, Stoye JP, Bishop KN, Taylor IA (2014). Structural basis of lentiviral subversion of a cellular protein degradation pathway. Nature.

[68] Schwefel D, Boucherit VC, Christodoulou E, Walker PA, Stoye JP, Bishop KN, Taylor IA (2015). Molecular determinants for recognition of divergent SAMHD1 proteins by the lentiviral accessory protein Vpx. Cell Host Microbe.

[69] Hwang TL, Shaka AJ (1995). Water suppression that works. excitation sculpting using arbitrary wave-forms and pulsed-field gradients. J Magn Reson Series A.

[70] Ordonez P, Bishop KN, Stoye JP, Groom HCT (2021). Analysis of SAMHD1 restriction by flow cytometry in human myeloid U937 cells. J Vis Exp.

[71] Mlcochova P, Caswell SJ, Taylor IA, Towers GJ, Gupta RK (2018). DNA damage induced by topoisomerase inhibitors activates SAMHD1 and blocks HIV-1 infection of macrophages. EMBO J.

[72] Sherman PA, Fyfe JA (1989). Enzymatic assay for deoxyribonucleoside triphosphates using synthetic oligonucleotides as template primers. Anal Biochem.

[73] Ferraro P, Franzolin E, Pontarin G, Reichard P, Bianchi V (2010). Quantitation of cellular deoxynucleoside triphosphates. Nucleic Acids Res.

