# Supplementary material for: Attenuation of reverse transcriptase facilitates SAMHD1 restriction of HIV-1 in cycling cells
Source: Retrovirology. 2023 May 1;20:5. doi: 10.1186/s12977-023-00620-z (PMC10150492; doi:10.1186/s12977-023-00620-z)
Supplement: Supplementary file 1 — Additional file 1: Table S1. T592 mutagenic primers for mammalian expression. Table S2. T592 mutagenic primers for E. coli expression. Figure S1. Vpx rescue assay outline. Figure S2. Testing SAMHD1 Nuclease activity. Figure S3. SAMHD1 T592 phospho-mutant panel. Figure S4. 1H NMR analysis of dNTP hydrolysis by SAMHD1 T592 mutants. Figure S5. Infectivity data for WT HIV and Q151N RT mutant infections in the presence of HU. Figure S6. SAMHD1 restriction in Jurkat cells infected with HIV-1 WT, RT V148I and Q151N mutants. Figure S7. Accumulation of viral reverse transcripts with time in U937 cells. [file 12977_2023_620_MOESM1_ESM.docx]

**Additional file Materials for :**

**Attenuation of reverse transcriptase facilitates SAMHD1 restriction of HIV-1 in cycling cells.**

Ming-Han C. Tsai^a^, Sarah J. Caswell^b*^, Elizabeth R. Morris^b*^, Melanie C. Mann^a^, Simon Pennell^c^, Geoff Kelly^d^, Harriet C.T. Groom^a^, Ian A. Taylor^b^ and Kate N. Bishop^a#^

1. **Additional file 1:** Table S1. T592 mutagenic primers for mammalian expression.
2. **Additional file 1:** Table S2. T592 mutagenic primers for *E. coli* expression.
3. **Additional file 1:** Figure S1. Vpx rescue assay outline.
4. **Additional file 1:** Figure S2. Testing SAMHD1 Nuclease activity.
5. **Additional file 1:** Figure S3. SAMHD1 T592 phospho-mutant panel.
6. **Additional file 1:** Figure S4. ^1^H NMR analysis of dNTP hydrolysis by SAMHD1 T592 mutants.
7. **Additional file 1:** Figure S5. Infectivity data for WT HIV and Q151N RT mutant infections in the presence of HU.
8. **Additional file 1:** Figure S6. SAMHD1 restriction in Jurkat cells infected with HIV-1 WT, RT V148I and Q151N mutants.
9. **Additional file 1:** Figure S7. Accumulation of viral reverse transcripts with time in U937 cells.

**Additional file 1: Table S1. T592 mutagenic primers for mammalian expression**

| T592 Mutant | Primer Direction | Sequences (5’🡪3’)* |
| --- | --- | --- |
| V | Forward | GTGATCGCCCCTCTGATC**GTC**CCTCAGAAAAAAGAGTG |
|  | Reverse | CACTCTTTTTTCTGAGG**GAC**GATCAGAGGGGCGATCAC |
|  | SAMHD1 592 (5’🡪3’) | ***^†^**Synthetic Fragment from ClaI (3,118) to SecII (3,895) in pLgateway_SAMHD1IRESYFP (5’🡪3’) |
| I | ATC | GATTCAATCGATACAATGATCACCGACGCCTTTCTGAAGGCCGACGACTA CATCGAGATCACCGGGGCTGGCGGCAAGAAGTACAGAATCAGCACCGCCA TCGACGACATGGAAGCCTACACCAAGCTGACCGACAACATCTTCCTGGAA ATCCTGTACAGCACCGACCCCAAGCTGAAGGACGCCAGAGAGATCCTGAA ACAGATCGAGTACCGGAACCTGTTCAAATACGTGGGCGAGACACAGCCCA CCGGCCAGATCAAGATCAAGAGAGAGGACTACGAGAGCCTGCCCAAAGAG GTGGCCAGCGCCAAGCCTAAGGTGCTGCTGGACGTGAAGCTGAAAGCCGA GGATTTCATCGTGGACGTGATCAACATGGACTACGGCATGCAGGAAAAGA ACCCCATCGACCACGTGTCCTTCTACTGCAAGACCGCCCCCAACCGGGCC ATCCGGATCACCAAGAATCAGGTGTCACAGCTGCTGCCCGAGAAGTTCGC CGAGCAGCTGATTCGGGTGTACTGCAAAAAGGTGGACCGGAAGTCCCTGT ACGCCGCCAGACAGTACTTCGTGCAGTGGTGCGCCGACCGGAACTTCACC AAGCCTCAGGACGGCGACGTGATCGCCCCTCTGATC**NNN**CCTCAGAAAAA AGAGTGGAACGACAGCACCAGCGTGCAGAACCCCACCAGACTGAGAGAGG CCAGCAAGAGCAGAGTGCAGCTGTTCAAGGACGACCCTATGTGAACCCAG CTTTCTTGTACAAAGTGGTGATTCGACGGTACCGCGGGATTCA |
| C | TGC |  |
| L | CTG |  |
| K | AAG |  |
| S | TCC |  |

*Mutagenized nucleotides at SAMHD1 592 position are shown in bold; ^†^Flanking sequence for ClaI-SecII fragment is highlighted in grey.

**Additional file 1: Table S2. T592 mutagenic primers for *E. coli* expression**

| T592 substitution | Primer | Sequence* |
| --- | --- | --- |
| A | T592A-F | GTTATAGCCCCACTCATA**G**CACCTCAAAAAAAGGAATG |
|  | T592A-R | CATTCCTTTTTTTGAGGTG**C**TATGAGTGGGGCTATAAC |
| D | T592D-F | GTTATAGCCCCACTCATA**GAT**CCTCAAAAAAAGGAATGG |
|  | T592D-R | CCATTCCTTTTTTTGAGG**ATC**TATGAGTGGGGCTATAAC |
| E | T592E-F | GTTATAGCCCCACTCATA**GA**ACCTCAAAAAAAGGAATGG |
|  | T592E-R | CCATTCCTTTTTTTGAGGT**TC**TATGAGTGGGGCTATAAC |
| V | T592V-F | GTTATAGCCCCACTCATA**GT**ACCTCAAAAAAAGGAATGG |
|  | T592V-R | CCATTCCTTTTTTTGAGGT**AC**TATGAGTGGGGCTATAAC |
| I | T592I-F | GTTATAGCCCCACTCATAA**TT**CCTCAAAAAAAGGAATGG |
|  | T592I-R | CCATTCCTTTTTTTGAGG**AA**TTATGAGTGGGGCTATAAC |
| C | T592C-F | GTTATAGCCCCACTCATA**TGT**CCTCAAAAAAAGGAATGG |
|  | T592C-R | CCATTCCTTTTTTTGAGG**ACA**TATGAGTGGGGCTATAAC |
| L | T592L-F | GTTATAGCCCCACTCATA**TT**ACCTCAAAAAAAGGAATGG |
|  | T592L-R | CCATTCCTTTTTTTGAGGT**AA**TATGAGTGGGGCTATAAC |
| K | T592K-F | GTTATAGCCCCACTCATAA**A**ACCTCAAAAAAAGGAATGG |
|  | T592K-R | CCATTCCTTTTTTTGAGGT**T**TTATGAGTGGGGCTATAAC |
| S | T592S-F | GTTATAGCCCCACTCATA**T**CACCTCAAAAAAAGGAATG |
|  | T592S-R | CATTCCTTTTTTTGAGGTG**A**TATGAGTGGGGCTATAAC |

*Sequences are 5’ – 3’, mutagenized nucleotides are shown in bold.

**
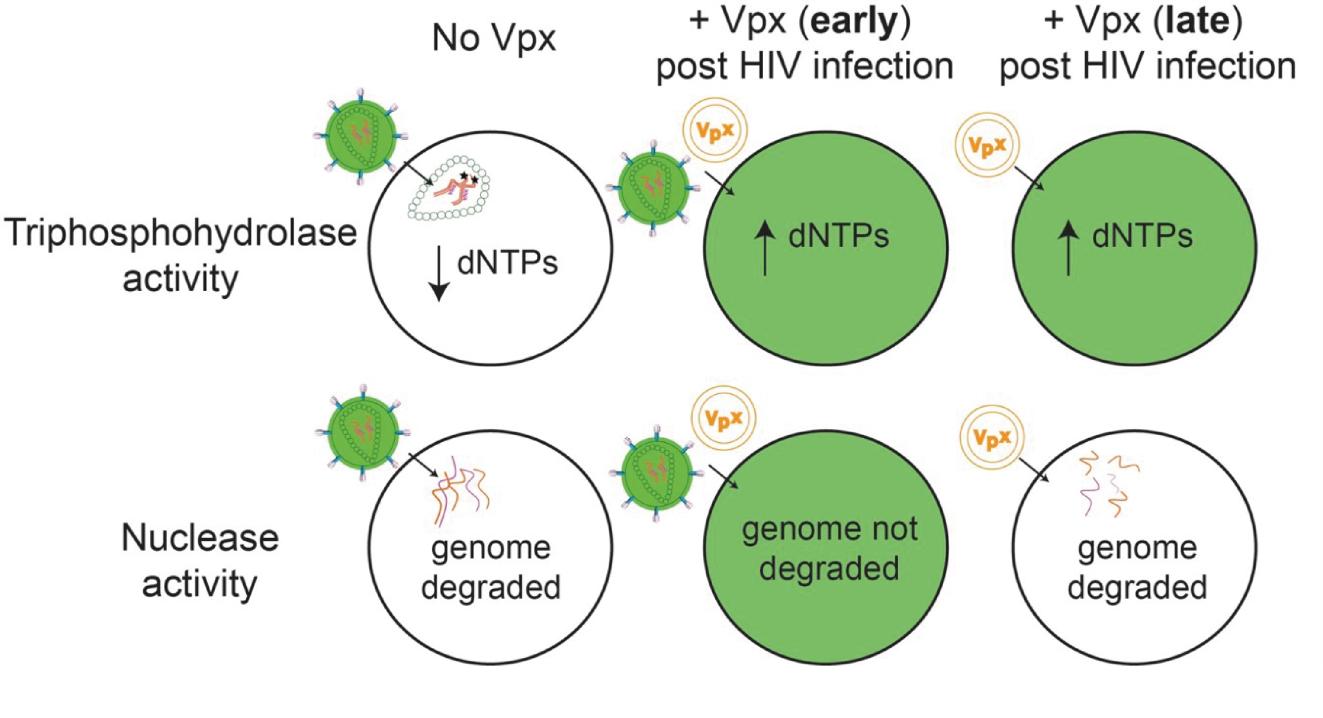
**

**Additional file 1: Figure S1. Outline of Vpx rescue assay**.

Schematic of Vpx rescue assay used to probe the mechanism of HIV-1 restriction by SAMHD1. Circles represent U937 cells expressing WT SAMHD1. Cells are infected with HIV-1-GFP VLPs and Vpx is introduced at different times post infection (early or late). In the absence of Vpx, SAMHD1 restricts viral replication and no GFP expression is observed. If Vpx is introduced early post infection, then it overcomes any SAMHD1 restriction mechanism, replication proceeds, and the cells express GFP. If Vpx is added at very late time points post infection, then the outcome depends upon the mechanism of SAMHD1 restriction: If triphosphohydrolase activity is responsible for restriction, then as soon as SAMHD1 is degraded, dNTP levels can rise sufficiently for replication to proceed. However, if the viral genome is degraded by nuclease activity, then adding Vpx at later timepoints would fail to rescue infection.

**
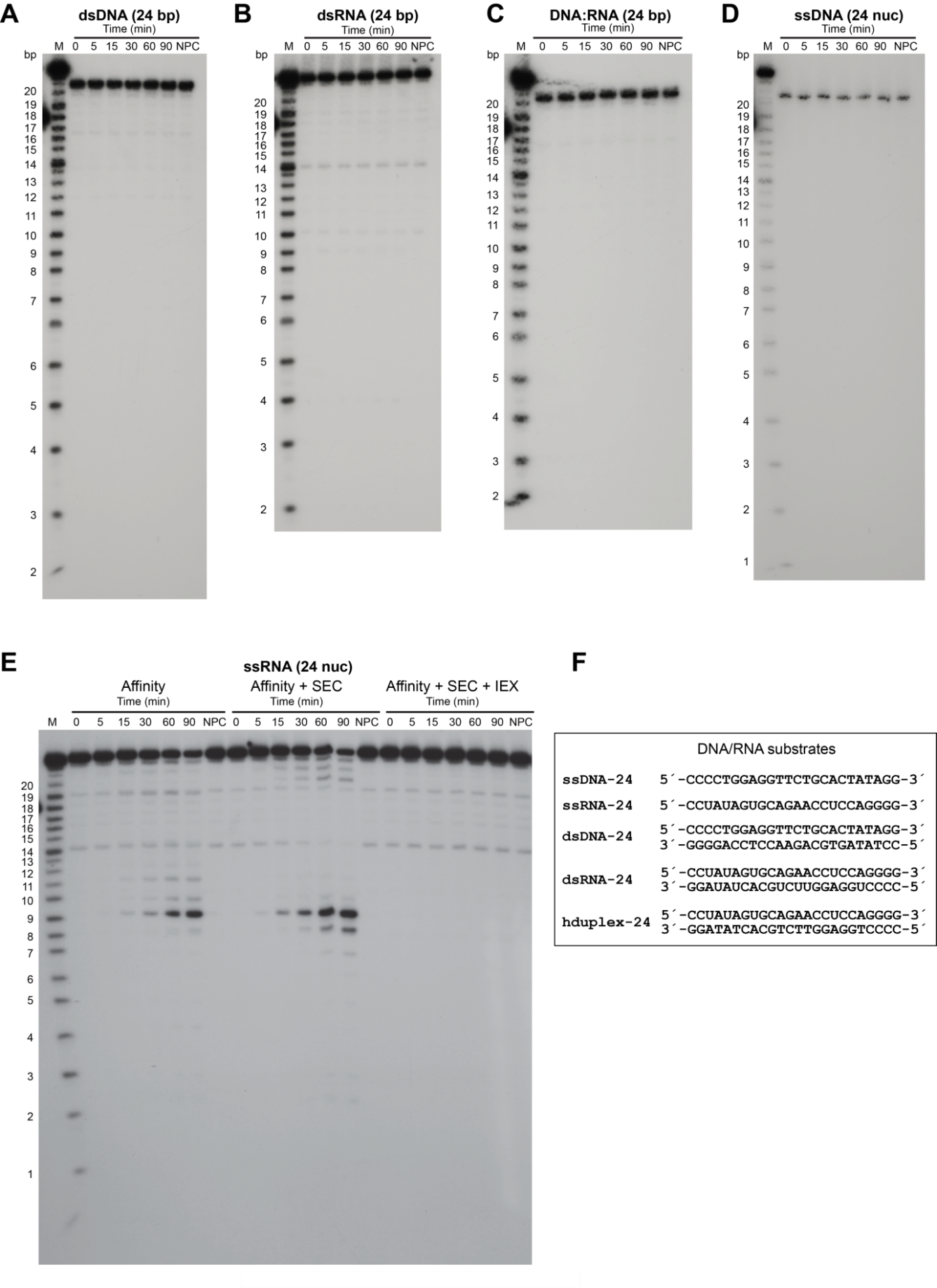
**

**Additional file 1: Figure S2. Testing SAMHD1 Nuclease activity**.

Urea denaturing gel electrophoresis analyses of time courses of SAMHD1 incubation (0-90 minutes) with different 24mer nucleic acid substrates. M indicates nucleotide size marker, length of each oligonucleotide in the ladder is indicated; NPC is no SAMHD1 protein control. SAMHD1 was purified by successive phases (Affinity, StrepTactin-agarose; SEC, Superdex 200; IEX, Mono-Q). Purified SAMHD1 was incubated with (**A**) double-stranded DNA, (**B**) double-stranded RNA, (**C**) DNA:RNA heteroduplex, (**D**) single-stranded DNA, (**E**) single-stranded RNA. In (**E**), SAMHD1 from each stage of purification was tested for ssRNA nuclease activity. (**F**) Sequences of 24mers employed in nuclease assays.


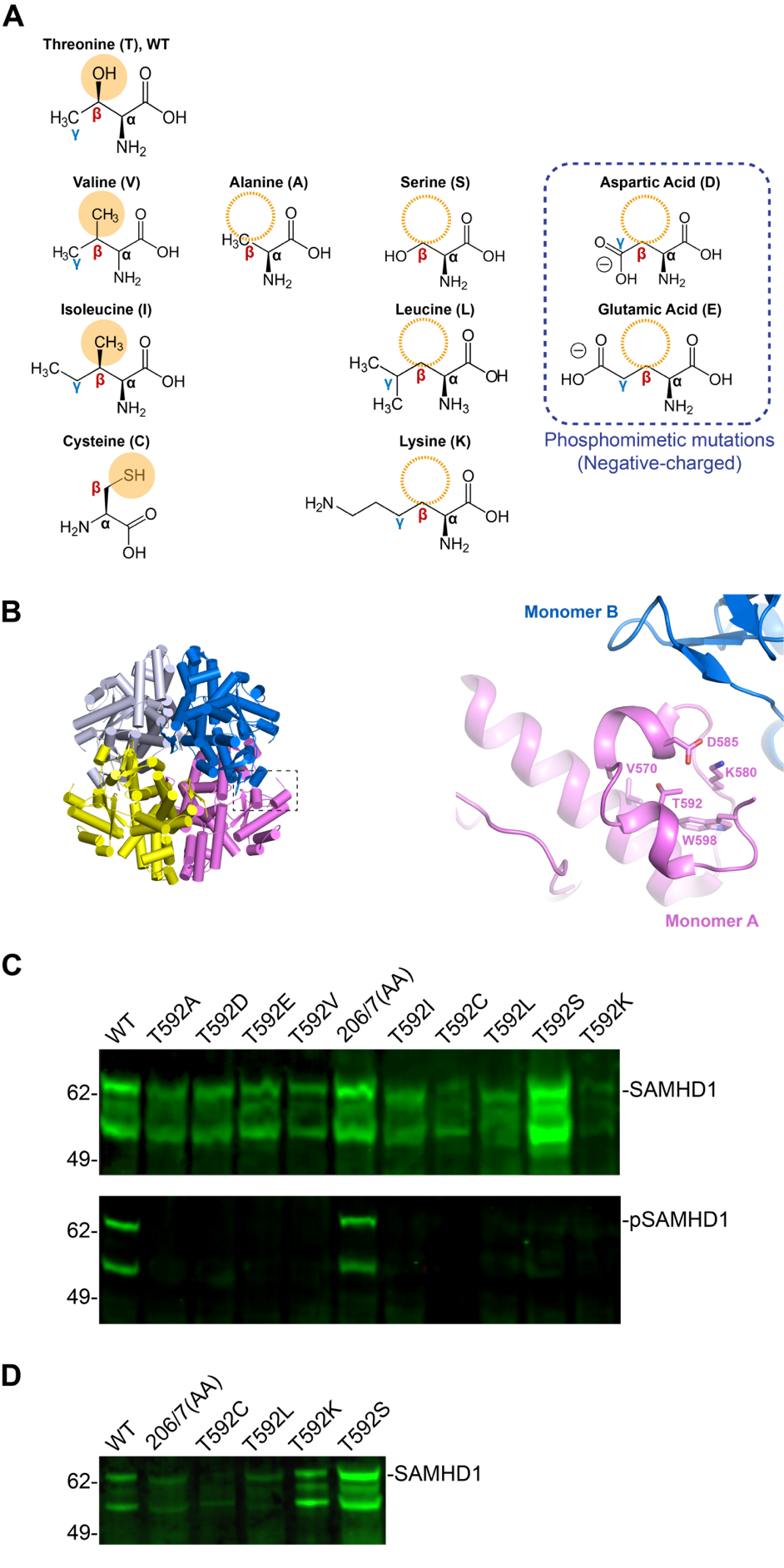


**Additional file 1: Figure S3. SAMHD1 T592 phospho-mutant panel.**

**(A)** Schematic view of amino acids substitutions in the SAMHD1 T592 mutant panel. Orange circles highlight residues with a bulky or branched hydrophobic group at the Cβ position. Open circles highlight residues with no branching at Cβ. Phosphomimetic mutants T592D and T592E that introduce a negative charge are boxed. **(B)** (Left) crystal structure of the SAMHD1 tetramer (PDB ID: 4BZC) the four monomers are shown in cartoon representation, coloured grey, blue, yellow, and magenta. (Right) structure of the SAMHD1 C-terminal lobe around the T592 phosphorylation site, boxed region in the left panel. In monomer A (magenta), the T592 γ-hydroxyl group is in close proximity to the side chain of D585 and the γ-methyl packs against W598 and the aliphatic portion of K580. Introduction of a phosphoryl group at T592 prevents these interactions and disrupts the packing with other protomers (blue) to destabilise the SAMHD1 tetramer. Amino acid substitutions that maintain the hydrophobic group at the branched Cβ position (V, I, C) promote tetramer stability **(C)** Expression and phosphorylation of WT SAMHD1, catalytically dead HD206/7AA and T592 mutants following transduction of cycling U937 cells with VLPs co-expressing YFP-SAMHD1 or mutants. Protein expression/phosphorylation was analysed by immunoblotting with antibodies to either SAMHD1 (top panel) or phospho-SAMHD1 (bottom panel). The molecular weights of size markers are indicated on the left. **(D)** Expression of WT SAMHD1, catalytically dead HD206/7AA and selected T592 mutants in differentiated U937 cells. Protein expression was analysed by immunoblotting with an antibody to SAMHD1. The molecular weights of size markers are indicated on the left.


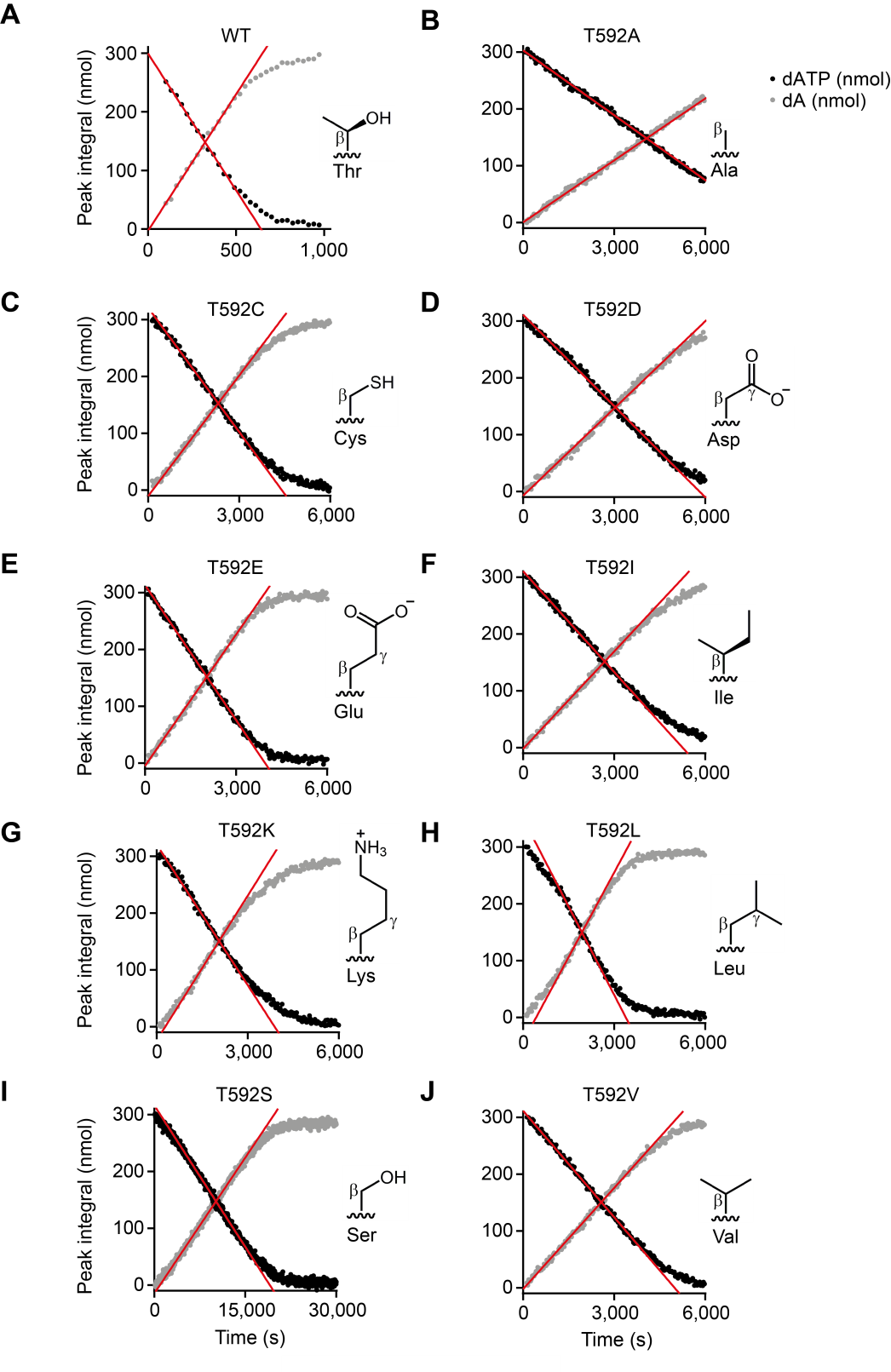


**Additional file 1: Figure S4. ^1^H NMR analysis of dNTP hydrolysis by SAMHD1 T592 mutants**

^1^H NMR analysis of GTP-activated hydrolysis of dATP by (**A**) WT SAMHD1, (**B**) T592A, (**C**) T592C, (**D**) T592D, (**E**) T592E, (**F**) T592I, (**G**) T592K, (**H**) T592L, (**I**) T592S, (**J**) T592V SAMHD1 mutants. Data were recorded for SAMHD1 hydrolysis reactions containing 1 μM SAMHD1, 0.2 mM GTP AL1-activator and dATP. In each panel, the integral of resolved substrate (black circle) and product (grey circle) peak resonances are plotted against time. Rates of hydrolysis were determined from slopes (red lines) derived from the data measured in the linear phase of the reaction, presented in Table 1.

**
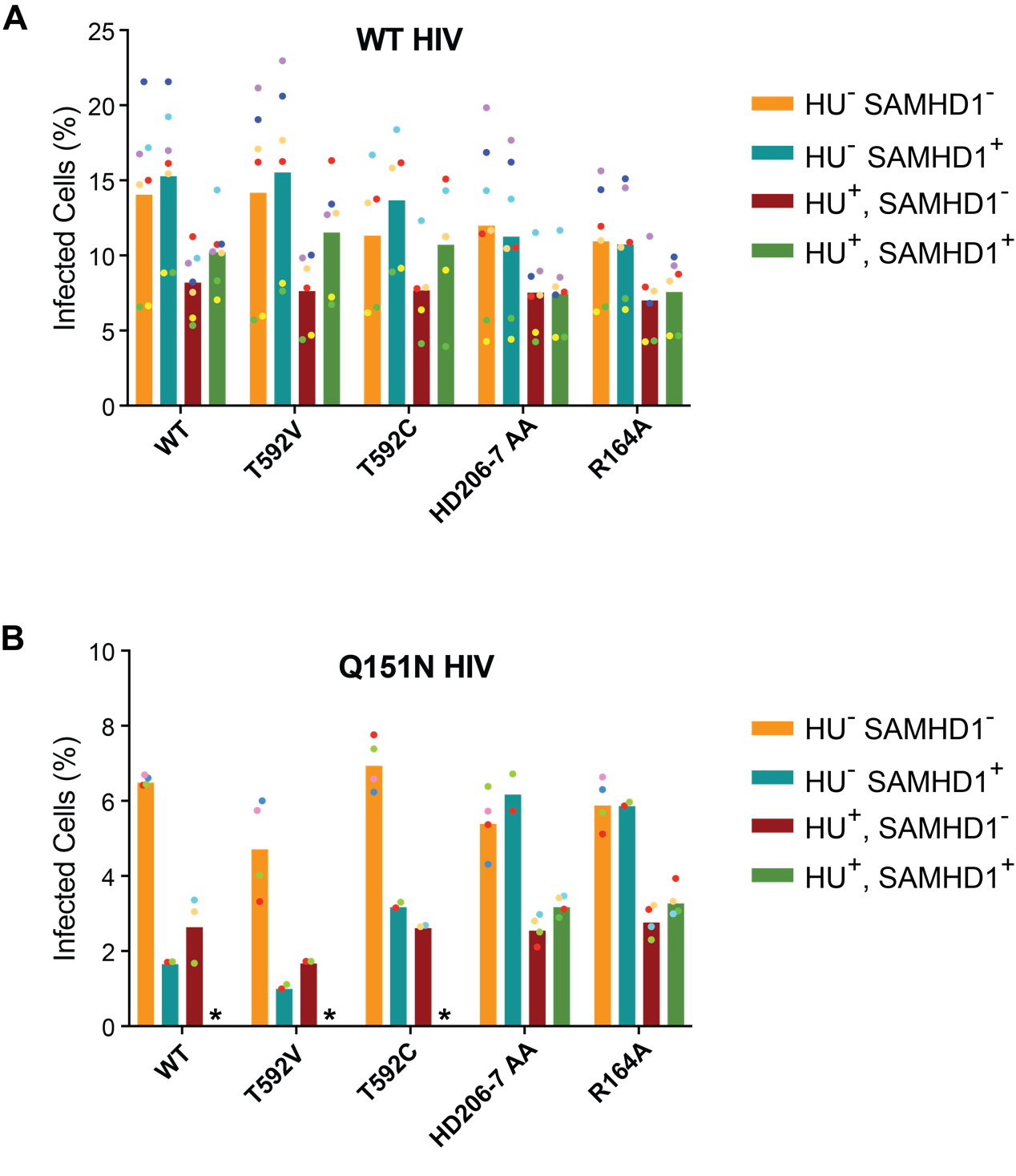
**

**Additional file 1: Figure S5. Infectivity data for WT HIV and Q151N RT mutant infections in U937 cells in the presence of HU (related to Figure 3F and Figure 4E)**

Cells were transduced with a bicistronic construct expressing WT SAMHD1 or the indicated SAMHD1 mutant and YFP, followed by infection with HIV-GFP carrying (**A**) WT RT or (**B**) RT Q151N. Cells were treated with 1 mM HU for 72 h (red and green bars) or untreated (orange and cyan bars), after which they were harvested and analysed by flow cytometry. The percentage of infected (GFP+) cells was calculated for YFP (SAMHD1) positive (cyan and green bars) and YFP negative (orange and red bars) populations. Points represent data from individual experiments and the bars show the mean. *Very few double positive cells (GFP+YFP+ <0.05%) were counted for WT SAMHD1, T592V, and T592C HU-treated cells.

**
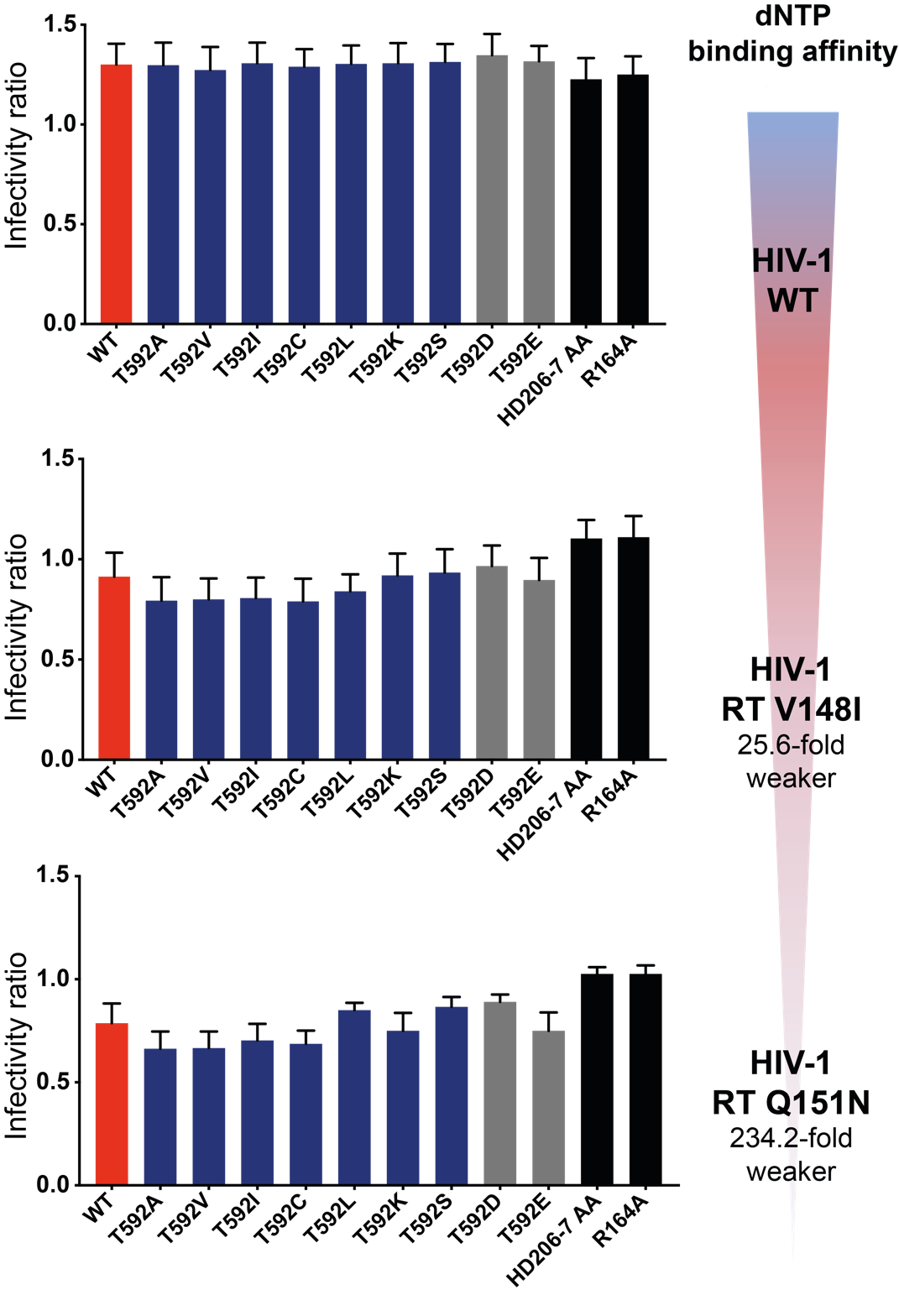
**

**Additional file 1: Figure S6. SAMHD1 restriction of HIV-1 WT, RT V148I and Q151N mutants in Jurkat cells**.

Cells were transduced with a bicistronic construct expressing WT SAMHD1 or the indicated mutant and YFP, followed by infection with HIV-GFP carrying WT RT (top panel), RT V148I (middle panel), or RT Q151N (bottom panel) mutants. Cells were harvested after 72 h and analysed by flow cytometry. The infectivity ratio was calculated by dividing the percentage of GFP positive cells in the YFP (SAMHD1) positive population by the percentage of GFP positive cells in the YFP (SAMHD1) negative population. The bars show the average of three biological repeats and error bars represent the SD. The T592 mutants, phosphomimetic mutants, and inactive negative controls are shown in blue, grey, and black, respectively. The relative dNTP binding affinity of each RT is indicated schematically on the right.


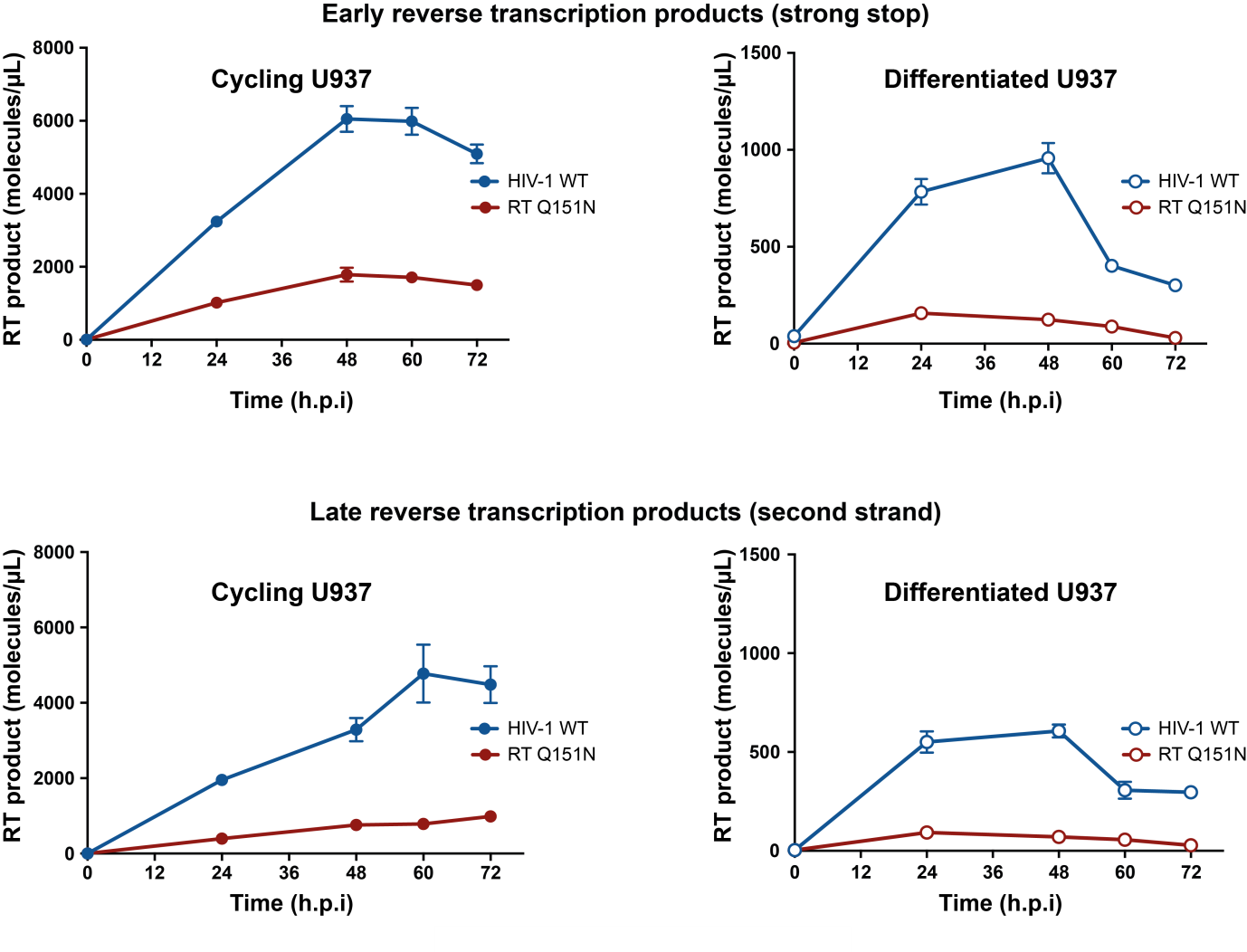


**Additional file 1: Figure S7. Time dependence of viral reverse transcript accumulation in U937 cells**.

Cycling (solid circles) and differentiated (open circles) U937 cells expressing SAMHD1 were infected with HIV-1-GFP, WT RT (blue) or RT-Q151N (red). Cells were harvested at the indicated time post infection and total DNA extracted. Early (strong stop, upper panels) and late (second strand, lower panels) reverse transcription products were quantified by qPCR. Each point represents the mean of three technical repeats and error bars are the SD.
